# Machine learning integrating immune-related cells, immunoglobulins, and immuno-inflammatory markers identifies risk factors for pulmonary comorbidities in ankylosing spondylitis: stratified analyses by chronic obstructive pulmonary disease and pulmonary nodules

**DOI:** 10.3389/fimmu.2026.1855064

**Published:** 2026-08-19

**Authors:** Shuang Ji, Jiayun Ping, Long Cheng, Tianqi Zhang, Zhangwei Huang, Lianzi Wang, Chao Cheng, Xu Zhang

**Affiliations:** 1 Department of Respiratory and Critical Care Medicine, First Affiliated Hospital of Anhui Medical University, Hefei, China; 2 Department of Health Management Center, First Affiliated Hospital of Anhui Medical University, Hefei, China; 3 Department of Oncology, First Affiliated Hospital of Anhui Medical University, Hefei, China; 4 First Clinical College and First Affiliated Hospital of Anhui Medical University, Hefei, China; 5 Department of Clinical Laboratory, First Affiliated Hospital of Anhui Medical University, Hefei, China; 6 Department of Respiratory and Critical Care Medicine, Anhui Provincial Chest Hospital, Hefei, China; 7 Department of Epidemiology and Biostatistics, School of Public Health, Anhui Medical University, Hefei, China

**Keywords:** ankylosing spondylitis, chronic obstructive pulmonary disease, comorbidity, machine learning, pulmonary diseases, pulmonary nodules

## Abstract

**Objective:**

Ankylosing spondylitis (AS) and pulmonary diseases (PD) comorbidities accelerates functional decline and increases disease burden, but risk stratification and identification of these comorbidities are lacking. This study developed machine learning (ML) models based on immune-related cells and immuno-inflammatory markers to identify risk factors for pulmonary comorbidities in AS.

**Methods:**

Demographic characteristics, immuno-inflammatory indices (e.g., neutrophils, erythrocyte sedimentation rate [ESR], C-reactive protein [CRP], immunoglobulin G, neutrophil-to-lymphocyte Ratio [NLR], platelet-to-neutrophil Ratio [PNR], pan-immune-inflammation value [PIV]) were measured and calculated for each patient. Feature selection was performed via least absolute shrinkage and selection operator (LASSO). Four ML algorithms, including decision tree, random forest, XGBoost, and support vector Machine (SVM), were developed and evaluated to distinguish between AS and AS+PD. Subgroup analyses were conducted for pulmonary nodules (PN) and chronic obstructive pulmonary disease (COPD), and sensitivity analysis was performed using unimputed data.

**Results:**

363 AS patients were enrolled, comprising 193 AS alone and 170 AS+PD patients (including 123 with PN and 47 with COPD). LASSO regression identified age, neutrophils, IgG, ESR, CRP, NLR, PNR, and PIV as key predictors for AS+PD. In the overall population, the SVM model demonstrated superior generalization and resistance to overfitting, whereas the random forest and XGBoost showed overfitting. In subgroup analyses, XGBoost emerged as the optimal classifier for AS+PN and AS+COPD, with good calibration and net clinical benefit. Sensitivity analyses confirmed the robustness and consistency of these findings.

**Conclusion:**

AS patients with pulmonary comorbidities exhibit distinct immune-related cells and immuno-inflammatory markers (especially neutrophils, IgG, NLR, and PIV). Of the four ML models, SVM showed best overall generalization, whereas XGBoost performed excellently in PN and COPD subgroups. These findings support the integration of immuno-inflammatory markers with ML algorithms to improve identification of current status of pulmonary complications for pulmonary comorbidities in AS.

## 1 Introduction

Ankylosing spondylitis (AS), a chronic, progressive, autoimmune spondylarthritis, primarily affects the axial skeleton and sacroiliac joints (1). The pathophysiology of AS is characterized by dysregulation of innate and adaptive immune responses, with the IL-23/IL-17 axis playing a central role in driving enthesitis and new bone formation (1, 2). Although axial and peripheral joint involvement are the hallmark clinical features of AS, extra-articular manifestations (EAMs) are highly prevalent and significantly contribute to increased disease burden and functional impairment (3–5). Among these EAMs, pulmonary disease (PD) involvement represents one of the most clinically consequential yet frequently underrecognized complications of AS (5, 6).

Pulmonary complications in AS include a spectrum of heterogeneous pathologies, including pulmonary bullae, bronchiectasis, pulmonary nodules (PN), and chronic obstructive pulmonary disease (COPD) (6). Epidemiological data indicate that approximately 30% of AS patients experience clinically significant respiratory symptoms, whereas high-resolution computed tomography (HRCT) reveals parenchymal lung abnormalities in 40%-90% of asymptomatic patients (7). Despite these severe clinical consequences, risk stratification and early identification of AS patients prone to pulmonary comorbidities remain challenging in routine clinical practice. Currently, there is a lack of effective and readily accessible biomarkers or predictive models for monitoring pulmonary status of AS patients.

Systemic inflammation serves as the core link between joint activity and extra-articular organ damage in AS. Although traditional markers such as the erythrocyte sedimentation rate (ESR) and C-reactive protein (CRP) are widely used to monitor disease activity of AS, their predictive value for specific organ complications is limited (8). Therefore, composite indices derived from complete blood count, such as the neutrophil-to-lymphocyte ratio (NLR), platelet-to-lymphocyte ratio (PLR), and the more recently proposed pan-immune inflammation value (PIV), have garnered attention (9, 10). These indicators represent the interactions between innate and adaptive immunity and may provide greater insight into the immunopathological mechanisms underlying disease progression and comorbidity development than single biomarkers do (11–13). However, the specific utility of these composite biomarkers in distinguishing AS patients with PD from those without PD has not been fully evaluated.

In recent years, machine learning (ML) models have emerged as important tools in disease diagnosis, prognosis prediction, and patient stratification (9, 14). In the field of AS research, a recent ML-based study demonstrated that the LightGBM framework effectively identifies distinct autoimmune comorbidity phenotypes in AS, highlighting the significant potential of ML in early risk stratification and clinical decision support in real-world studies (15). Nevertheless, the comparative performance and generalizability of different ML algorithms in the use of routine immune and inflammatory data to distinguish AS patients with specific pulmonary comorbidities remain to be systematically assessed.

Therefore, this study aims to provide a foundation for enhancing identification of current status of pulmonary complications and informing the comprehensive management strategies for pulmonary comorbidities of AS by integrating immuno-inflammatory profiles with ML computational modeling.

## 2 Materials and methods

### 2.1 Study design

This cross-sectional study was conducted at the First Affiliated Hospital of Anhui Medical University between January 1st, 2020 and March 31st, 2026. The study protocol was approved by the Clinical Research Ethics Committee of the First Affiliated Hospital of Anhui Medical University (No. 2022185), and the requirement for written informed consent was waived owing to the retrospective nature of the study.

### 2.2 Study participants

Initially, a total of 870 newly diagnosed patients with AS who had not received prior standard treatment (i.e., no prior use of biologics, glucocorticoids, or immunosuppressants) were enrolled according to the modified New York criteria (1984) (16). Participants meeting the following criteria were excluded: (1) with other autoimmune or rheumatic diseases (n=105); (2) with hepatitis A virus, hepatitis B virus, or hepatitis C virus infection (n=67); (3) with tuberculosis (n=54); (4) with multiple metabolic diseases (n=145); (5) with other malignant diseases (e.g., tumors, n=45); and (6) aged <18 years or ≥85 years (n=91). Based on the exclusion criteria, a total of 363 eligible patients were ultimately included in the analysis.

**Figure 1:**
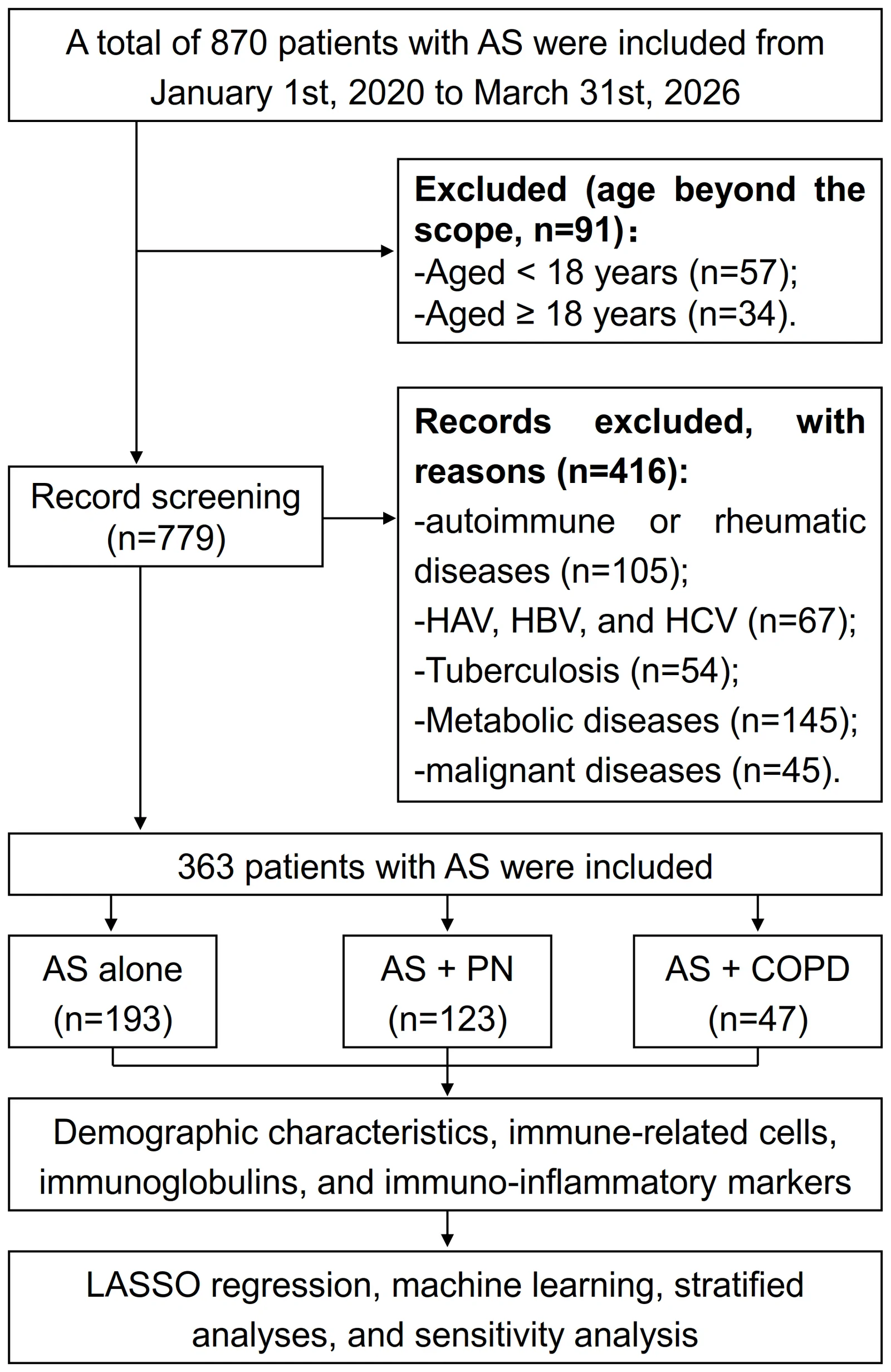
Flowchart of participants screening.

A composite pulmonary disease outcome (COPD and PN) was used as a pragmatic screening endpoint because pulmonary complications in AS are frequently asymptomatic and not routinely screened. A positive screening result triggered further confirmatory examinations, including HRCT and pulmonary function tests. In addition, both COPD and PN share common mechanisms based on inflammation associated with the IL-23/IL-17 axis, and prespecified subgroup analyses were carried out to evaluate disease-specific patterns. Accordingly, the final study cohort was stratified into two groups based on the presence or absence of PD (defined as documentation of at least one of the aforementioned pulmonary diagnoses in the medical records): COPD was defined according to the GOLD criteria (post-bronchodilator FEV_1_/FVC < 0.70), and PN was diagnosed by chest HRCT. The diagnosis of PN was established based on the chest HRCT performed closest to the study enrollment date (within the preceding 6 months), which revealed a discrete, well-circumscribed radiographic opacities ≤ 3 cm in diameter. Of the 363 patients, 170had confirmed pulmonary comorbidities (i.e., AS+PD group), including 123 cases with PN (AS+PN group) and 47 cases with COPD (AS+COPD group); no PD patient had both conditions concurrently. The remaining 193 AS patients without PN constituted the AS group. The detailed flowchart of participant screening is shown in **Figure 1**.

### 2.3 Data collection and measurements

Clinical and demographic characteristics, including age, sex, and smoking history (such as smoking status and average daily cigarette consumption), were recorded. Fasting venous blood samples (3 ml) were collected in the early morning, and complete blood cell count analysis was performed within two hours using an automatic hematology analyzer (Mindray, Shenzhen, China) (14). The institutional central laboratory conducted laboratory data assessments, which included ESR, CRP, rheumatoid factor (RF), immunoglobulin G (IgG), immunoglobulin A (IgA), immunoglobulin M (IgM), complement component 3 (C3), neutrophils, lymphocytes, monocytes, eosinophils, basophils, and platelets, using standard automated biochemical analyzer (14). In addition, Bath Ankylosing Spondylitis Disease Activity Index (BASDAI) was used for evaluating the disease activity, and Bath Ankylosing Spondylitis Functional Index (BASFI) was used for assessing the bodily functions.

### 2.4 Inflammatory-immune biomarkers

The following composite immuno-inflammatory indices were calculated for each participant (9, 10, 17): (1) NLR, calculated as neutrophil divided by lymphocyte; (2) PLR, calculated as platelet divided by lymphocyte; (3) lymphocyte-to-monocyte ratio (LMR), calculated as lymphocyte divided by monocyte; (4) platelet-to-neutrophil ratio (PNR), calculated as platelet divided by neutrophil; (5) C-reactive protein-to-lymphocyte ratio (CLR), calculated as CRP divided by lymphocyte; and (6) PIV, calculated as (neutrophil × platelet × monocyte)/lymphocyte.

### 2.5 Feature selection

Prior to model development, candidate predictors were screened for multicollinearity. First, variables that demonstrated significant differences between the AS group and the AS+PD group were included in the collinearity diagnostics (14, 18). The variance inflation factor (VIF) and tolerance value for each variable were calculated. A threshold of VIF < 10 and tolerance > 0.1 was applied to confirm the absence of problematic multicollineakrity. Second, variables were further analyzed using least absolute shrinkage and selection operator (LASSO) regression. LASSO is widely used for screening variables because of its ability to handle multicollinearity (18). A nomogram constructed via multivariate regression was subsequentlyemployed to visualize the significant variables. Finally, based on the independent risk factors, ML models were developed to predict the risk of PD in patients with AS.

### 2.6 ML models

The dataset comprising 363 patients was randomly divided into a training set and a test set at a ratio of 70%:30%, i.e., the training set consisted of 254 patients (approximately 135 AS and 119 AS+PD), while the test set included 109 patients (approximately 58 AS and 51 AS+PD). The training set was used for all model fitting and hyperparameter optimization and the test set was used for final model evaluation. This procedure was applied identically to all four ML models: (1) Decision Tree (DT), a non-parametric method that recursively partitions the feature space based on information gain or Gini impurity; (2) Random Forest (RF), an ensemble learning method that constructs a multitude of decision trees and aggregates their predictions via majority voting; (3) eXtreme Gradient Boosting (XGBoost), a gradient boosting ensemble algorithm that sequentially builds weak learners, with each subsequent model focusing on correcting the errors of its predecessor; and (4) Support Vector Machine (SVM) with a Radial Basis Function (RBF) kernel, which maps input features into a high-dimensional space to identify the optimal separating hyperplane with the maximal margin (9, 18, 19). A fixed random seed of 123 and default threshold of 0.5 was used for all ML models. Given the relatively small sample size of the AS+COPD group (n = 47), a 5-fold cross-validation was employed as a sensitivity analysis to evaluate the performance of the ML models. In addition, SHapley Additive exPlanations (SHAP) analysis was used for interpreting ML model predictions and quantifying feature importance.

### 2.7 Subgroup analysis

To construct an inclusive screening framework, the primary analysis considered any form of pulmonary disease as a composite outcome, but pre-specified subgroup analyses were conducted for the two most prevalent subtypes, PN and COPD, to explore disease-specific associations. First, a subgroup analysis was conducted to compare AS alone with AS+PN among the 170 patients in the AS+PD cohort. Second, another independent subgroup analysis was performed to compare AS alone with AS+COPD. The analytical workflow, including univariate screening, collinearity diagnostics, LASSO regression, and the development of four ML models, was independently repeated for each subgroup analysis.

### 2.8 Sensitivity analysis

To mitigate their excessive influence on regression models, where unaddressed missing values can disproportionately affect coefficient estimates and model fit, multiple imputation was utilized to handle data with a missing ratio of less than 10%, as previously published article (14). In addition, to evaluate the robustness of the study findings and the potential impact of data imputation on model performance and variable selection, a sensitivity analysis was conducted. In the sensitivity analysis, all analytical workflows were repeated using the original unimputed dataset (i.e., a complete-case analysis), and consistency between the primary and sensitivity analyses was assessed.

### 2.9 Statistical analysis

Prior to statistical analysis, an assessment for outliers was conducted on continuous variables, as previously published methodology (14). Potential univariate outliers were identified using boxplots and the interquartile range (IQR) method, with values below Q1-1.5 × IQR or above Q3 + 1.5 × IQR being flagged. All flagged outliers further subjected to a dual check for data entry errors and clinical plausibility. As the identified outliers were considered biologically plausible (e.g., extreme but possible laboratory values), they were retained in the primary analysis.

The normality of continuous variables was assessed using the Kolmogorov-Smirnov test. Data comparisons between the two groups (i.e., AS+PD *vs.* AS, AS+PN *vs.* AS, and AS+COPD *vs.* AS) were performed using the Mann-Whitney U test. Categorical variables (i.e., sex, smoking history) were described using sample size and percentage, and were compared using the chi-square test. Bonferroni correction was conducted based on the classification of variables (i.e., the threshold for significant significance was defined as *P* < 0.05 divided by the number of subsets). Variables that showed significant differences after the Bonferroni correction in the univariate analysis were included in LASSO regression to identify independent risk factors for the comorbidity of AS and PD/PN/COPD. The prediction results were subsequently visualized using a nomogram. Furthermore, four ML models were developed based on the identified significant variables. The predictive performance of all models was evaluated using receiver operating characteristic (ROC) curves and the corresponding area under the curve (AUC) values, accuracy, sensitivity, specificity, precision, positive predictive value (PPV), and negative predictive value (NPV). Model fit was assessed using calibration curve, recall value, and F1 score. To prevent data leakage, all data preprocessing steps, including imputation and LASSO-based feature selection, were performed exclusively within the training set. Parameters derived from the training set were applied to the test set. The test set remained completely unseen during model training and hyperparameter tuning. In addition, decision curve analysis (DCA) curve was used to quantify the net benefit at different threshold probabilities, providing a visual assessment of the clinical utility offered by a predictive model. This approach aims to assist clinical decision-makers in selecting and implementing appropriate testing strategies. All statistical analyses were performed using R software, with *P* < 0.05 considered statistically significant.

## 3 Results

### 3.1 Demographic characteristics, immune-related cells, and immuno-inflammatory biomarkers

Initially, 870 potential participants were identified in this study. After excluding 91 participants whose ages were beyond the range and 416 participants with other comorbidities, a total of 363 patients were ultimately included. The cohort comprised 193 patients with AS alone and 170 patients with the comorbidities of AS and pulmonary disease (AS+PD, including 123 patients with PN 47 patients with COPD) (**Figure 1**).

The median age in the AS+PD group was significantly higher than that in the AS group (Z = -5.628, *P* < 0.001, **Table 1**), whereas no significant differences were observed between the two groups in smoking history, BASDAI, and BASFI (*P* > 0.05, **Table 1**). Furthermore, levels of immune cells and immuno-inflammatory biomarkers, including ESR, CRP, IgG, IgM, neutrophils, NLR, PLR, CLR, and PIV, were significantly elevated in the AS+PD group compared to the AS group, whereas levels of lymphocytes, LMR, and PNR were lower (**Table 1**). No statistically significant differences were observed in sex, RF, IgA, C3, monocytes, eosinophils, basophils, and platelets between the two groups (*P* > 0.05, **Table 1**).

**Table 1:** Clinical characteristics and immuno-inflammatory markers of the two groups.

Overall	AS (n=193)	AS+PD (n=170)	Z	*P*	*P*_Bonferroni_
Demographic/clinical characteristics					0.05/4 = 0.0125
Sex (Male, %)	151 (78.2)	130 (76.5)	0.162^*^	0.688	NSD
Age (Years)	34.0 (27.8, 43.0)	40.5 (31.0, 52.0)	-5.628	<0.001	SD
Smoking (Yes, %)	112 (58.0)	108 (63.5)	1.144^*^	0.285	NSD
ADCC (Count)	19.0 (0, 25.0)	22.0 (0, 25.0)	-1.175	0.240	NSD
Inflammatory markers					0.05/3 = 0.0167
ESR (mm/h)	22.5 (8.0, 41.0)	31.0 (15.0, 57.0)	-5.882	<0.001	SD
RF (IU/mL)	8.0 (6.0, 10.0)	8.0 (7.0, 10.0)	-1.818	0.069	NSD
CRP (mg/L)	8.1 (3.7, 23.4)	17.8 (7.3, 38.2)	-6.434	<0.001	SD
Immunological indicators					0.05/4 = 0.0125
IgG (g/L)	13.0 (12.0, 15.0)	14.0 (12.0, 17.0)	-3.476	0.001	SD
IgA (g/L)	3.0 (2.0, 4.0)	3.0 (2.0, 4.0)	-0.383	0.701	NSD
IgM (g/L)	1.0 (1.0, 2.0)	1.0 (1.0, 22.0)	-4.118	<0.001	SD
C3 (g/L)	1.0 (1.0, 1.0)	1.0 (1.0, 1.0)	-0.014	0.989	NSD
Blood immune cells					0.05/6 = 0.008
NEU (× 10^9/L)	4.1 (3.2, 5.0)	4.4 (3.6, 5.6)	-3.387	0.001	SD
LYM (× 10^9/L)	2.1 (1.6, 2.6)	1.9 (1.4, 2.4)	-4.15	<0.001	SD
MON (× 10^9/L)	0.43 (0.35, 0.53)	0.44 (0.36, 0.56)	-1.066	0.286	NSD
EOS (× 10^9/L)	0.12 (0.08, 0.22)	0.12 (0.07, 0.20)	-0.336	0.737	NSD
BAS (× 10^9/L)	0.03 (0.02, 0.05)	0.03 (0.02, 0.05)	-1.012	0.311	NSD
PLT (× 10^9/L)	270.0 (228.8, 321.0)	277.0 (227.3, 336.0)	-0.374	0.708	NSD
Disease activity/function score					0.05/2 = 0.025
BASDAI (cm)	1.68 (0.78, 2.44)	1.71 (0.84, 2.54)	-0.891	0.373	NSD
BASFI (cm)	1.0 (0.9, 1.8)	1.1 (0.8, 1.8)	-0.359	0.719	NSD
Composite inflammatory markers					0.05/6 = 0.008
NLR	1.9 (1.5, 2.4)	2.4 (1.8, 3.2)	-6.526	<0.001	SD
PLR	128.1 (98.4, 166.1)	145.9 (113.3, 191.8)	-4.541	<0.001	SD
LMR	4.9 (3.9, 6.0)	4.1 (3.3, 5.4)	-5.308	<0.001	SD
PNR	66.2 (54.1, 83.7)	59.2 (49.0, 80.3)	-3.354	0.001	SD
CLR	4.2 (1.63, 12.6)	8.7 (3.4, 19.9)	-6.724	<0.001	SD
PIV	224.4 (140.2, 357.9)	272.0 (188.0, 464.5)	-4.743	<0.001	SD

*Chi-square value; ADCC, Average daily cigarette consumption; BASDAI, Bath Ankylosing Spondylitis Disease Activity Index; BASFI, Bath Ankylosing Spondylitis Functional Index; ESR, Erythrocyte sedimentation rate; RF, Rheumatoid factor; CRP, C-reactive protein; IgG, Immunoglobulin G; IgA, Immunoglobulin A; IgM, Immunoglobulin M; C3, Complement component 3; NEU, Neutrophil; LYM, Lymphocyte; MON, Monocyte; EOS, Eosinophil; BAS, Basophil; PLT, Platelet; NLR, Neutrophil-to-Lymphocyte Ratio; PLR, Platelet-to-Lymphocyte Ratio; LMR, Lymphocyte-to-Monocyte Ratio; PNR, Platelet-to-Neutrophil Ratio; CLR: C-reactive Protein-to-Lymphocyte Ratio; PIV, Pan-Immune-Inflammation Value.

*P*_Bonferroni_, 0.05 divided by the number of subsets; SD, Significant difference; NSD, Not significant difference.

### 3.2 Collinearity diagnostics, LASSO regression, and ROC analysis

**Figure 2:**
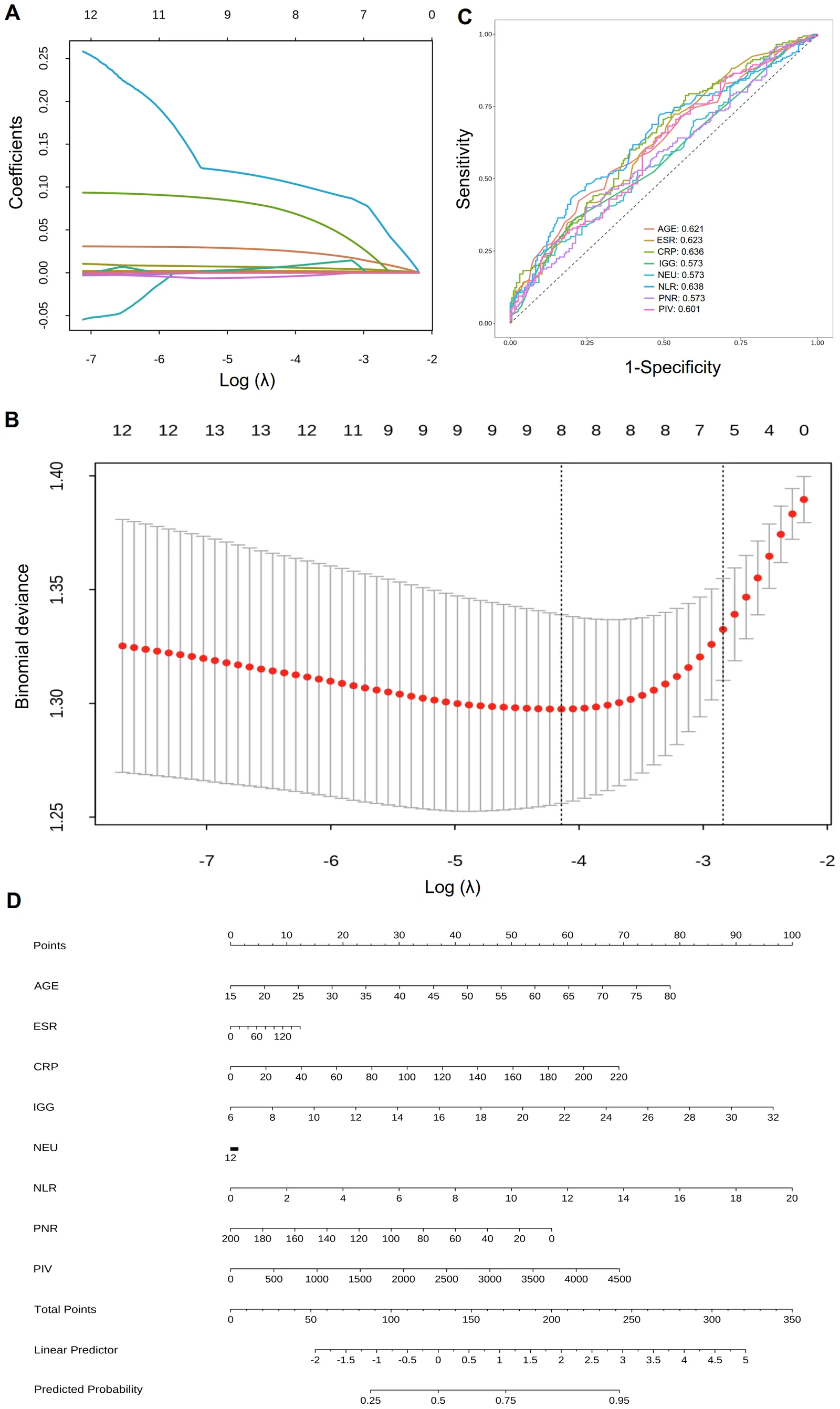
Feature selection and predictive results. **(A)** LASSO regression selected parameters. **(B)** LASSO regression profiles of selected 8 parameters. **(C)** Predictive value of a single parameters. **(D)** Nomogram for predicting the probability of AS+PD.

Multicollinearity assessment including 13 significant variables (age, ESR, CRP, IgG, IgM, neutrophils, lymphocytes, NLR, PLR, LMR, PNR, CLR, and PIV), the highest VIF was 9.679 (for CLR), and tolerances exceeded 0.1, indicating no multicollinearity among these variables (**Supplementary Table 1**). Candidate predictors of 13 variables were further included in a LASSO regression, and age, ESR, CRP, IgG, neutrophils, NLR, PNR, and PIV were ultimately left in the model (**Figures 2A–C**). These findings were also visualized using a nomogram (**Figure 2D**). All variables demonstrated modest discriminative ability, with AUC values ranging from 0.573 to 0.638 (**Supplementary Table 2**; **Figure 2C**).

### 3.3 ML performance in differentiating between AS and AS combined with PD

**Figure 3:**
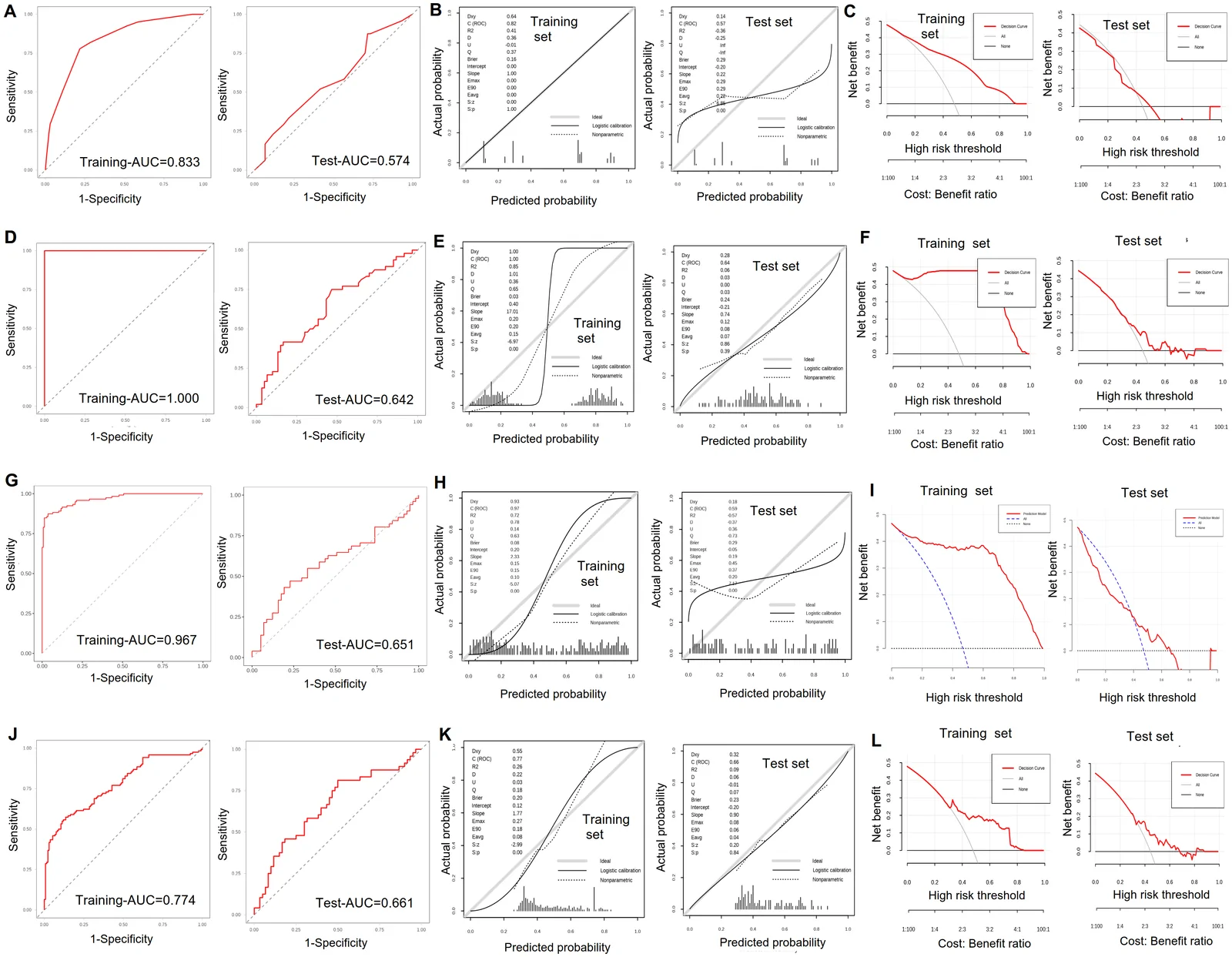
ML performance in estimating the risk of PD in AS patients. **(A)** AUC, **(B)** Calibration curve, and **(C)** DCA curve of the decision tree model. **(D)** AUC, **(E)** Calibration curve, and **(F)** DCA curve of the random forest model. **(G)** AUC, **(H)** Calibration curve, and **(I)** DCA curve of the XGBoost model. **(J)** AUC, **(K)** Calibration curve, and **(L)** DCA curve of the SVM model.

Four ML models based on eight indicators (Age, ESR, CRP, IgG, neutrophils, NLR, PNR, and PIV) were employed to distinguish between AS and AS+PD (**Table 2**; **Figures 3A–L**; **Supplementary Figures 1A–H**). The random forest model demonstrated high discriminative ability (sensitivity = 0.992, specificity = 1.000, accuracy = 0.996, and F1-score = 0.996; **Table 2**; **Figures 3D–F**), and the XGBoost achieved the highest values across all metrics (sensitivity = 1.000, specificity = 1.000, accuracy = 1.000, and F1-score = 1.000; **Table 2**; **Figures 3G–I**), indicating that both the XGBoost and random forest exhibited outstanding classification performance and possess capability for further clinical validation. However, the performance of the XGBoost, random forest, and decision tree models significantly declined on the test set (**Table 2**; **Figures 3A–I**), suggesting the presence of overfitting. Calibration curves revealed a deviation between predicted probabilities and actual incidence on the test set, and DCA indicated limited clinical net benefit for these three models (**Figures 3A–I**). The SVM was significantly superior to other methods in the terms of accuracy (0.716) and F1-score (0.670) (**Table 2**; **Figures 3A–I**). The SVM model achieved moderate consistency of performance (accuracy = 0.710, sensitivity = 0.820, specificity = 0.590, and F1-score = 0.747) on the training set (**Table 2**; **Figures 3J–L**). On the test set, the model performance remained stable (accuracy = 0.630, sensitivity = 0.70, specificity = 0.542, and F1-score = 0.677) (**Table 2**; **Figures 3J–L**). Therefore, the SVM demonstrated the best generalization capability and outstanding robustness, exhibiting the strongest resistance to overfitting.

**Table 2:** The performance of the four machine learning models in overall population and subgroups.

Categories	AUC		Accuracy		Sensitivity/recall		Specificity		F1 score		PPV/precision		NPV	
	Train	Test	Train	Test	Train	Test	Train	Test	Train	Test	Train	Test	Train	Test
Overall														
DT	0.833	0.574	0.780	0.556	0.782	0.583	0.779	0.521	0.788	0.593	0.741	0.483	0.815	0.612
RF	1.000	0.642	1.000	0.583	1.000	0.600	1.000	0.562	1.000	0.615	1.000	0.563	0.992	0.656
XGBoost	0.967	0.589	0.906	0.611	0.874	0.549	0.934	0.667	0.897	0.571	1.000	0.547	1.000	0.661
SVM	0.774	0.661	0.710	0.630	0.820	0.700	0.590	0.542	0.747	0.677	0.774	0.568	0.689	0.646
Pulmonary nodule														
DT	0.869	0.645	0.811	0.606	0.809	0.649	0.814	0.541	0.667	0.840	0.729	0.500	0.873	0.685
RF	1.000	0.704	1.000	0.691	1.000	0.842	1.000	0.459	1.000	0.768	1.000	0.654	1.000	0.706
XGBoost	0.987	0.747	0.937	0.677	0.885	0.500	0.971	0.789	0.917	0.545	0.591	0.500	0.263	0.713
SVM	0.804	0.700	0.725	0.702	0.956	0.947	0.360	0.324	0.810	0.794	0.838	0.800	0.703	0.684
COPD														
DT	0.863	0.728	0.875	0.833	0.955	0.900	0.571	0.500	0.924	0.900	0.769	0.500	0.894	0.900
RF	1.000	0.754	1.000	0.806	1.000	0.917	1.000	0.250	1.000	0.887	1.000	0.375	1.000	0.859
XGBoost	0.996	0.771	0.947	0.789	0.727	0.357	1.000	0.895	0.842	0.400	0.850	0.912	NA	1.000
SVM	0.880	0.771	0.821	0.833	1.000	1.000	0.143	<0.001	0.899	0.909	1.000	0.000	0.816	0.833

AUC, Area under curve; DT, Decision tree; RF, Random forest; SVM, Support vector machine; COPD, Chronic obstructive pulmonary disease; PPV, Positive predictive value; NPV, Negative predictive value; NA, Not available.

### 3.4 Subgroup analysis of comorbidity of AS and PN

**Figure 4:**
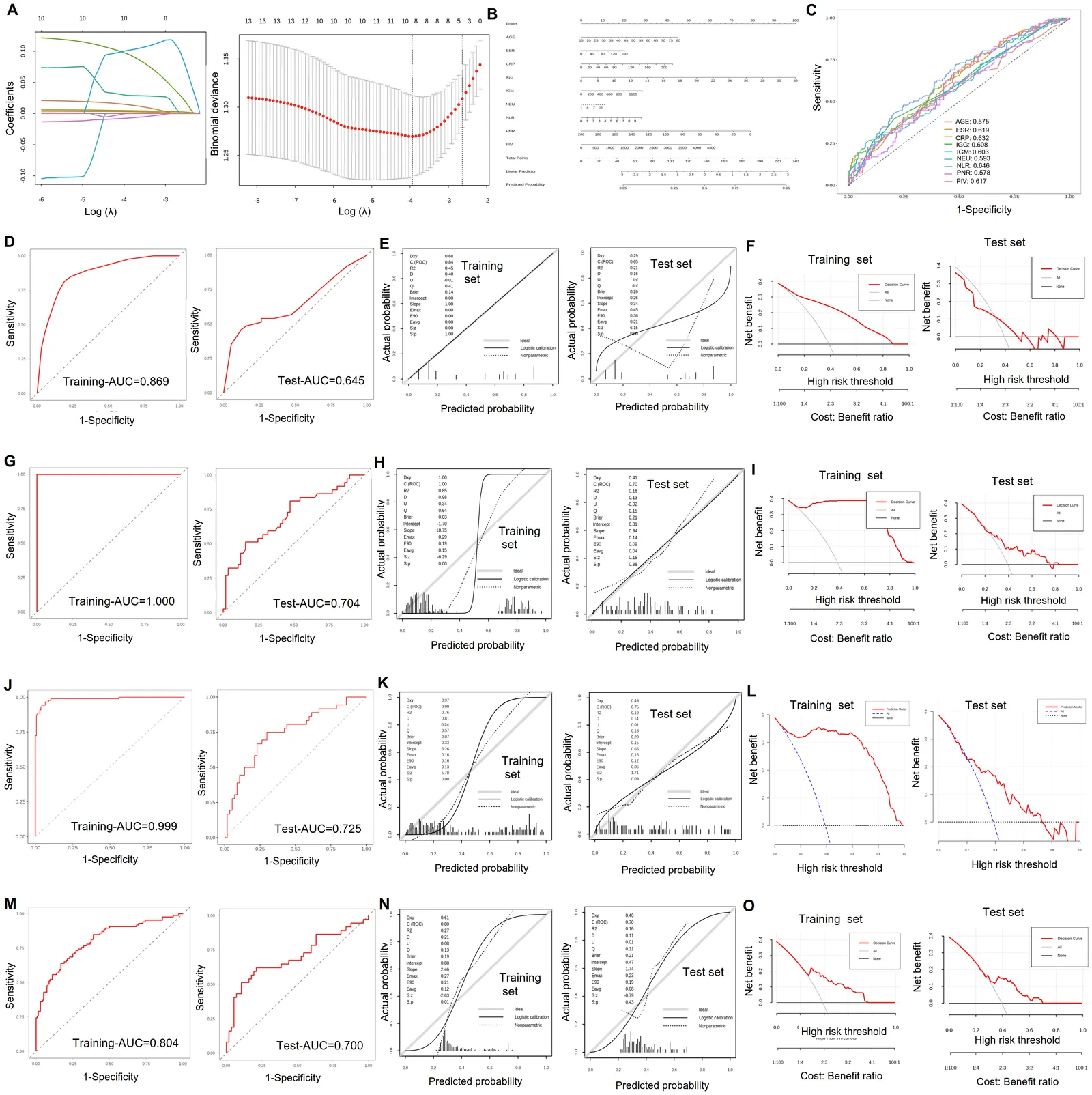
LASSO regression and ML performance in estimating the risk of PN in AS patients. **(A)** LASSO regression selected 9 parameters. **(B)** Nomogram for predicting the probability of AS+PN. **(C)** Predictive value of a single parameter. **(D)** AUC, **(E)** Calibration curve, and **(F)** DCA curve of the decision tree model. **(G)** AUC, **(H)** Calibration curve, and **(I)** DCA curve of the random forest model. **(J)** AUC, **(K)** Calibration curve, and **(L)** DCA curve of the XGBoost model. **(M)** AUC, **(N)** Calibration curve, and **(O)** DCA curve of the SVM model.

Of the 170 patients with comorbid AS and PD, 123 patients had AS combined with PN. On the basis of the findings of univariate analysis (**Supplementary Table 3**), LASSO regression and ROC analysis identified age, ESR, CRP, IgG, IgM, neutrophils, NLR, PNR, and PIV as independent risk factors in AS patients with PN (**Supplementary Table 4**; **Figures 4A–C**; **Supplementary Figures 2A–H**).

Of the four ML models trained and validated on the AS+PN dataset, XGBoost demonstrated the best generalization ability, achieving the highest AUC on the test set (**Table 2**; **Figures 4D–O**). The decision tree and random forest models exhibited excellent performance metrics including accuracy, sensitivity, specificity, precision, and F1 score, on the training set (especially random forest showing perfect fit; **Table 2**; **Figures 4D–I**). However, their performances declined significantly on the test set, indicating overfitting. Calibration curves and DCA further revealed a discrepancy between the predicted probabilities and the actual event rates on the test set, with limited clinical net benefit (**Figures 4E, F, H, I**). In contrast, the XGBoost model had balanced performances (accuracy = 0.677, specificity = 0.789 on the test set), and the highest discriminative ability (AUC = 0.747) (**Table 2**; **Figures 4J–L**). The calibration curve of the XGBoost model indicated good overall calibration on the test set (**Figure 4K**), and decision curve analysis further confirmed that the model provided positive net benefit within a threshold probability range of 0.1–0.6 (**Figure 4L**), suggesting potential clinical utility. The SVM model showed high sensitivity but very low specificity (**Table 2**; **Figures 4M–O**), indicating a strong ability to identify positive samples but a high rate of false positives for negative samples. Therefore, the XGBoost is recommended as the optimal model for pulmonary nodule classification.

### 3.5 Subgroup analysis of comorbidity of AS and COPD

**Figure 5:**
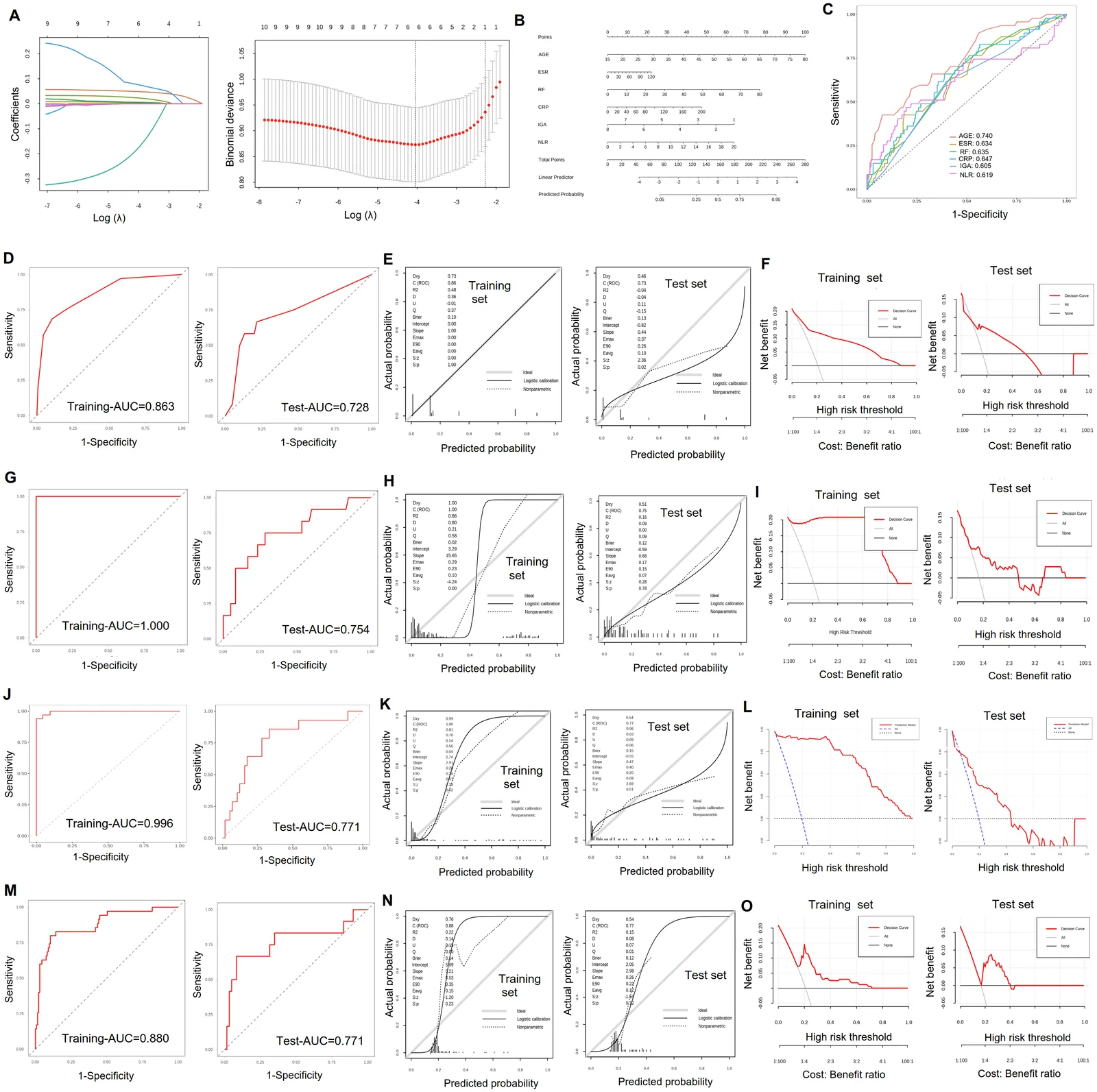
LASSO regression and ML performance in estimating the risk of COPD in AS patients. **(A)** LASSO regression selected 8 parameters. **(B)** Nomogram for predicting the probability of AS+COPD. **(C)** Predictive value of a single parameter. **(D)** AUC, **(E)** Calibration curve, and **(F)** DCA curve of the decision tree model. **(G)** AUC, **(H)** Calibration curve, and **(I)** DCA curve of the random forest model. **(J)** AUC, **(K)** Calibration curve, and **(L)** DCA curve of the XGBoost model. **(M)** AUC, **(N)** Calibration curve, and **(O)** DCA curve of the SVM model.

In the AS+PD group, 47 patients were AS combined with COPD. LASSO regression and ROC analysis identified age, ESR, RF, CRP, IgA, and NLR as independent risk factors in AS patients with COPD based on the univariate analysis results (**Supplementary Tables 5**, **6**; **Figures 5A–C**; **Supplementary Figures 3A–H**).

Next, we systematically evaluated the performance of four ML models for distinguishing between AS and AS+COPD (**Table 2**; **Figures 4D–O**). The decision tree model demonstrated good discriminative ability on the training set (accuracy = 0.875, sensitivity = 0.955, specificity = 0.571, F1 score = 0.924); its performance slightly declined in the test set but remained practical (**Table 2**; **Figures 5D, E**). Calibration and DCA further supported good calibration and clinical utility of decision tree model on the training set, although the degree of calibration decreased on the test set (**Figures 5E, F**). The random forest model exhibited a perfect fit on the training set, but its performance significantly declined in the test set, indicating overfitting and limited generalizability (**Table 2**; **Figures 5G–I**). The XGBoost model demonstrated favorable discriminative ability on the test set (AUC = 0.771), with an accuracy of 78.9% (**Table 2**; **Figures 5J–L**). The calibration curve indicated that the predicted probabilities were largely consistent with the actual event rates in the test set, suggesting good model calibration (**Figure 5K**). DCA further confirmed that XGBoost model provided a positive net clinical benefit within a threshold probability range of approximately 10%–50% (**Figure 5L**). Although the SVM model performed well on the training set, its precision was 0 and its specificity was approximately 0, indicating that the model classified all samples on the test set, regardless of whether they were from patients or healthy individuals, as positive (**Table 2**; **Figures 5M–O**). The SVM model completely lost its ability to identify positive cases, suggesting severe overfitting and class imbalance issues. Additionally, findings of 5-fold cross-validation also identified the XGBoost as the optimal model. It achieved the highest mean accuracy among all models (0.792, range: 0.688–0.875) and the highest mean specificity (0.811, range: 0.711–0.902). The model also showed stable fitting with no severe overfitting, as all R² values were non-negative (mean: 0.146) (**Supplementary Table 9**). Therefore, the XGBoost is recommended as the optimal model for COPD classification, whereas the decision tree model can serve as a viable alternative.

### 3.6 Sensitivity analysis results

**Figure 6:**
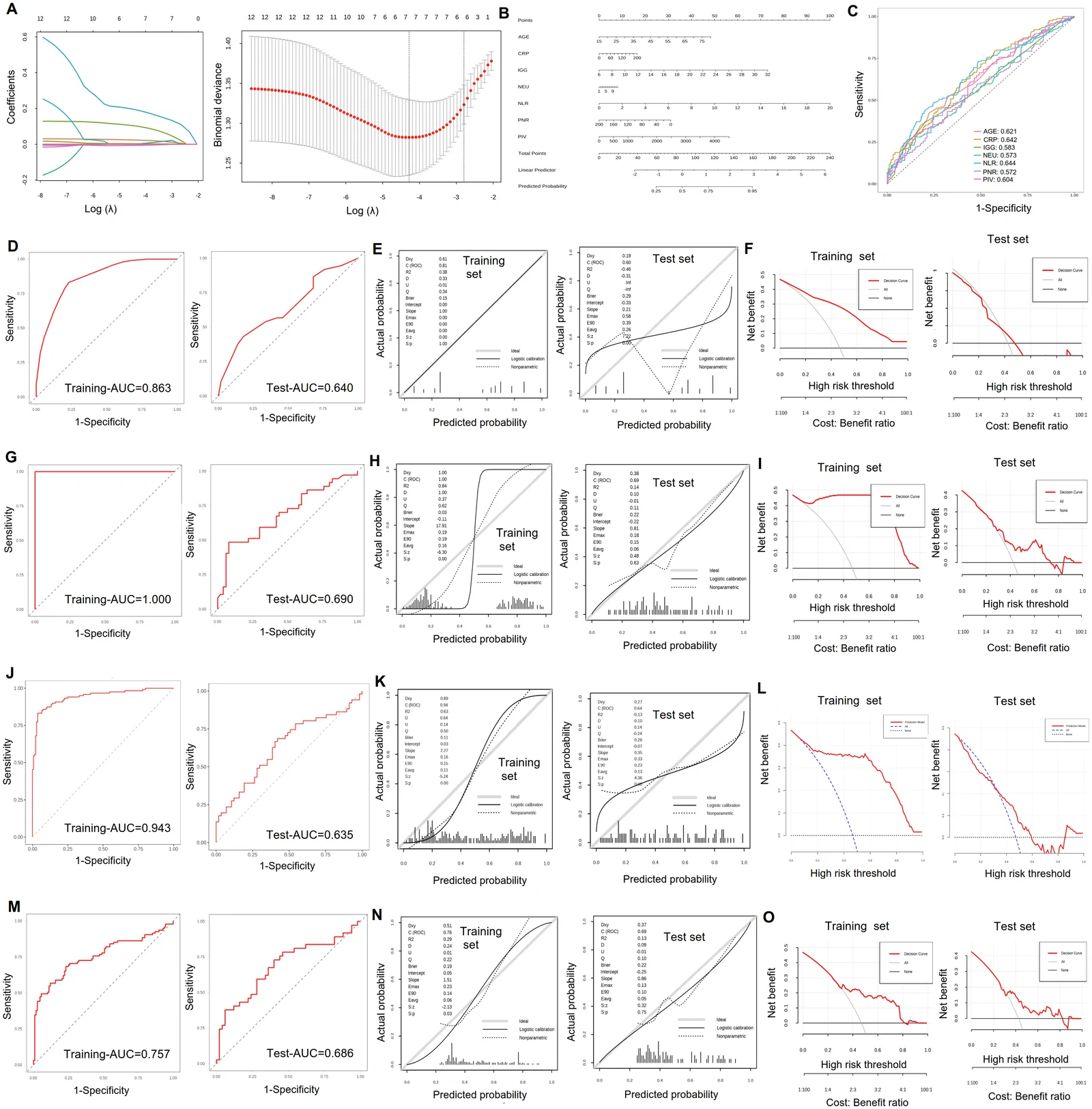
LASSO regression and ML performance in estimating the risk of PD in AS patients (sensitivity analysis). **(A)** LASSO regression selected 7 parameters. **(B)** Nomogram for predicting the probability of AS+COPD. **(C)** Predictive value of a single parameter. **(D)** AUC, **(E)** Calibration curve, and **(F)** DCA curve of the decision tree model. **(G)** AUC, **(H)** Calibration curve, and **(I)** DCA curve of the random forest model. **(J)** AUC, **(K)** Calibration curve, and **(L)** DCA curve of the XGBoost model. **(M)** AUC, **(N)** Calibration curve, and **(O)** DCA curve of the SVM model.

To validate the findings of the overall population, a sensitivity analysis was performed on the original data prior to imputation data. According to the sensitivity analysis, the median age of the AS+PD group was also significantly greater than that of the AS group (Z = -3.977, *P* < 0.001; **Table 1**). Furthermore, immune-related cells and immuno-inflammatory biomarkers exhibited the same trend as those in the first analysis (**Supplementary Table 7**). Of note, ESR was left in the preliminary analysis (input data) but was removed in the sensitivity analysis (non-input data) (**Figures 6A, B**; **Supplementary Figures 4A–H**), which is consistent with its correlation with other inflammatory markers and the collinearity elimination effect of LASSO. However, the performance of the SVM remained stable, indicating that model identification does not depend on any single biomarker. This discrepancy suggests that the sensitivity of variable selection to data preprocessing and reinforces the necessity of external validation to confirm the stability of the predictors. All variables demonstrated modest discriminative ability, with AUC values ranging from 0.605 to 0.740 (**Supplementary Table 8**; **Figure 6C**).

Four ML models were constructed on the sensitivity analysis dataset, and their fitting performances were evaluated by comparing metrics between the training set and the test set (**Table 3**; **Figures 6D–O**). The decision tree, random forest, and XGBoost models demonstrated excellent discriminative ability in the training set (especially the random forest model exhibited perfect fit, with accuracy, sensitivity, specificity, precision, and F1-score of 1.000; **Table 3**; **Figures 6D–L**). However, the performance of these three models significantly declined in the test set (**Table 3**; **Figures 6D–L**), indicating substantial overfitting and limited generalizability. The SVM model showed good discriminative ability in the training set, with a slight decrease in accuracy observed in the test set (**Table 3**; **Figures 6M–O**). Calibration curves indicated that the SVM models were well-calibrated in the training set, but might overestimate the probability for some intermediate- to high-risk individuals in the test set (**Figure 6N**). DCA demonstrated that the use of this model for clinical decision-making could yield net benefit within a threshold probability range of 10% to 60% (**Figure 6O**). Consequently, SVM was recommended as the optimal model for this sensitivity analysis. These findings are consistent with the results obtained from the first imputed dataset, indicating that our results are robust. The sensitivity analysis using complete-case (non-imputed) data yielded consistent findings, suggesting that missing data did not bias the results.

**Table 3:** The performance of the four machine learning models in overall population (sensitivity analysis).

Categories	AUC		Accuracy		Sensitivity		Specificity		Precision		Recall		F1 score		PPV		NPV	
	Train	Test	Train	Test	Train	Test	Train	Test	Train	Test	Train	Test	Train	Test	Train	Test	Train	Test
DT	0.863	0.640	0.798	0.552	0.769	0.540	0.832	0.568	0.838	0.628	0.769	0.540	0.802	0.581	0.760	0.477	0.838	0.628
RF	1.000	0.690	1.000	0.655	1.000	0.760	1.000	0.514	1.000	0.679	1.000	0.760	1.000	0.717	1.000	0.613	1.000	0.679
XGBoost	0.943	0.635	0.882	0.593	0.866	0.549	0.897	0.632	0.880	0.571	0.866	0.549	0.873	0.560	0.956	0.742	0.974	0.855
SVM	0.757	0.686	0.704	0.644	0.796	0.780	0.600	0.459	0.694	0.661	0.796	0.780	0.741	0.716	0.722	0.607	0.694	0.661

AUC, Area under curve; DT, Decision tree; RF, Random forest; SVM, Support vector machine; COPD, Chronic obstructive pulmonary disease; PPV, Positive predictive value; NPV, Negative predictive value.

## 4 Discussion

This study systematically investigated the differences in immune-related cells, immunoglobulins, and immuno-inflammatory profiles between AS alone and AS+PD and rigorously evaluated the performance of ML models in distinguishing these two patients. First, we found that the AS+PD group exhibited a significantly higher systemic inflammatory burden, as evidenced by elevated levels of conventional inflammation and immune-inflammation-related markers (neutrophils, ESR, CRP, IgG, and IgM) and dysregulation of composite indices (NLR, PLR, CLR, and PIV). Second, of the four ML models, the SVM demonstrated robust generalization capability in the overall population, whereas XGBoost was identified as the optimal classifier for subgroup classification involving PN and COPD. Finally, sensitivity analyses based on unimputed raw data confirmed the robustness of the associations and the performances of ML models, thereby enhancing the credibility of the study findings.

Systemic inflammation in AS is closely associated with EAMs, including those affecting the lungs (20). In this study, the AS+PD group was older and had significantly higher ESR, CRP, IgG, and IgM, which is consistent with the established knowledge linking EAMs to a high inflammatory burden. Pulmonary complications represent a significant EAM, with more than 30% of AS patients developing lung disease in the later stages of their disease course, reflecting the cumulative burden of chronic inflammation on lung parenchyma and airways (20, 21). Therefore, the development of non-invasive diagnostic tools based on immuno-inflammatory markers for the early identification of pulmonary lesions in AS is highly important. Furthermore, advanced age was identified as a significant demographic characteristic for AS+PD, indicating that enhanced pulmonary surveillance is warranted for older AS patients with persistently elevated inflammatory markers.

A novel contribution of this study is the evaluation of the role of composite hematological indices (e.g., the NLR, PLR, CLR, and PIV) in the comorbidity of AS and PD. Although NLR and PLR have been established as markers of disease activity in AS (22), the significance of novel indices such as PIV, CLR, and PNR in pulmonary comorbidities has not been fully investigated. Our finding that PIV was significantly elevated in the AS+PD group. By integrating multiple components of immune effector cells, PIV provides a more comprehensive reflection of the systemic immuno-inflammatory milieu than individual cell counts or two-component ratios and has been associated with adverse outcomes in autoimmune diseases (12, 13). Our data extend these findings by specifically implicating PIV dysregulation in the context of pulmonary comorbidity, suggesting that the combined activation of neutrophils and monocyte, thrombocytosis, and lymphopenia contribute to the systemic immune signature of pulmonary involvement in AS. However, elevated PIV in our AS+PD cohort may not be AS-specific but rather reflect a shared systemic immune-inflammatory pathway common to various autoimmune conditions with lung involvement. For example, elevated PIV has been described in other autoimmune diseases with pulmonary complications, such as rheumatoid arthritis-associated interstitial lung disease, suggesting that PIV likely reflects a shared state of enhanced innate immune activation rather than a disease-specific marker (12). Moreover, PIV is elevated in systemic sclerosis patients with interstitial lung disease compared to healthy controls, and novel inflammatory biomarkers like PIV may be superior to simple hematological parameters in reflecting inflammatory activity of connective tissue diseases accompanied by fibrotic lung involvement (23). Recent population-based studies also confirmed that elevated PIV is significantly associated with worse respiratory outcomes, including increased cough/sputum and declines in lung function (24). These findings further support that PIV reflects systemic inflammation affecting the lungs irrespective of the underlying autoimmune condition. In this study, AS+PD patients had elevated NLR, PLR, CLR, and PIV values and decreased LMR and PNR values, which is consistent with innate immune dysregulation andIL-23/IL-17 axis activation in extra-articular damage (25, 26). Hence, integrating readily available hematological parameters into composite indices can provide valuable insights into the systemic immune status associated with pulmonary comorbidities in AS.

The core objective of this study was to evaluate the efficacy of ML models based on immune-related cells, immunoglobulins, and immuno-inflammation-related indicators in distinguishing AS+PD from AS alone. Although the application of ML in autoimmune disease researched has expanded rapidly, overfitting and poor generalizability remain critical challenges (27, 28). In this study, our comparative analysis of four ML algorithms yielded insightful findings. Random forest exhibited near-perfect performance on the training set, highlighting its capacity to model complex interactions. However, its performance declined significantly on the test set, with substantial miscalibration and limited net clinical benefit. This discrepancy represents overfitting and poor generalizability, especially in subgroup analyses with smaller sample sizes (e.g., AS+COPD, n=47). In contrast, the SVM demonstrated superior robustness and generalizability in distinguishing AS from AS+PD, with consistent performance between the training and test sets (stable accuracy, sensitivity, and F1-score), suggesting that SVM has stronger resistance to overfitting and better generalization performance on datasets with moderate sample sizes and potential near-linear relationships (15). Furthermore, SVM maintained acceptable performance in sensitivity analysis, further validating its reliability as the preferred model for distinguishing between AS and AS+PD. However, the SVM model yielded an AUC of 0.661 on the test set, suggesting only moderate discriminatory performance, which may limit its clinical utility as a standalone tool, thus it should be regarded as a supplementary screening tool rather than a definitive diagnostic test. In addition, we emphasized that the primary advantage of this model in the present study lies in its superior generalization capability and robustness against overfitting, particularly in comparison with more complex models. In brief, our findings provide empirical support for the rational selection of ML models in medium-sized autoimmune disease datasets.

Subgroup analyses yielded more nuanced recommendations for model selection. In the AS+PN subgroup, XGBoost outperformed other models, achieving the highest AUC (0.747) on the test set with good calibration and positive net benefit across clinically relevant threshold probability ranges. This suggests that XGBoost can capture subtle nonlinear relationships specific to PN phenotype without succumbing to the overfitting observed in the overall analysis. In the AS+COPD subgroup, XGBoost again showed favorable discriminatory ability, although its performance was modest (AUC = 0.771), and the decision tree model provided a feasible and explainable appropriate alternative. SVM failed to identify positive cases in the AS+COPD subgroup, highlighting the vulnerability of margin-based classifiers to severe class imbalance and the necessity of matching modeling strategies with the specific data characteristics of each subgroup. These findings collectively argue against a “one-size-fits-all” approach to ML model in clinical researches, emphasizing that model selection (i.e., SVM is the recommended choice for the overall population, whereas XGBoost is preferred for subgroups.) must be tailored to the specific clinical question, data characteristics, and the trade-offs between sensitivity, specificity, and interpretability. In addition, given the limited sample size of the AS+COPD subgroup, these findings are considered exploratory and are intended solely for hypothesis generation. Therefore, external validation in larger cohorts is necessary prior to any clinical application.

This study has several strengths. First, it is the first to systematically describe and compare multiple composite immuno-inflammatory indices in the comorbidity of AS and PD (including PN and COPD). Second, a robust framework was constructed by comparing four ML algorithms, emphasizing resistance to overfitting, calibration, and net clinical benefit. Furthermore, sensitivity analyses further strengthened confidence in the robustness and reproducibility of findings. However, several limitations should be considered. First, the retrospective, single-center design of this study and the lack of independent external validation limit the generalizability of the findings to broader populations, and the performances of ML models may vary across different ethnic groups. Therefore, the proposed model of this study should be considered exploratory and not yet suitable for clinical application. Future research should conduct a prospective, multi-center study design and perform external validation to validate the current findings. Second, the sample size, especially the AS+COPD subgroup (n=47), was relatively modest. Third, prior to diagnosis, some patients may have received non-standard treatments, such as the administration of anti-inflammatory and analgesic medications, which could have altered their systemic inflammatory levels and thereby biased our results. Forth, due to the retrospective nature of the study, precise PN characteristics (size, texture, and number) could not be obtained from prior imaging reports and were thus not included in subgroup stratification. Future studies should investigate the potential impact of PN characteristics on disease outcomes. Finally, LASSO regression ignored potential interactions among different variables, which may lead to the omission of profound predictive patterns. Although the tree-based machine learning models employed in this study inherently possess the capability to model complex nonlinear interactions, future researches could incorporate interaction terms or adopt models specifically designed to detect interaction effects, thereby further enhancing predictive performance.

In conclusion, this study demonstrates that the comorbidity of AS and PD exhibit a distinct pattern of systemic inflammation and immune dysregulation, characterized by older age, elevated levels of inflammatory markers (ESR, CRP, and IgG), and imbalances in composite indices such as the NLR and PIV. ML models, especially SVM and XGBoost, can effectively utilize these immuno-inflammatory parameters to distinguish between AS and AS+PD. These findings highlight the potential of integrating immuno-inflammatory markers with rigorously validated ML methodologies to improve identification of current status of pulmonary complications and comprehensive management for pulmonary comorbidities in patients with AS.

## Data Availability

The original contributions presented in the study are included in the article/**Supplementary Material**. Further inquiries can be directed to the corresponding author.
