# Supplementary material for: Machine learning integrating immune-related cells, immunoglobulins, and immuno-inflammatory markers identifies risk factors for pulmonary comorbidities in ankylosing spondylitis: stratified analyses by chronic obstructive pulmonary disease and pulmonary nodules
Source: Front Immunol. 2026 Aug 19;17:1855064. doi: 10.3389/fimmu.2026.1855064 (PMC13533909; doi:10.3389/fimmu.2026.1855064)
Supplement: Supplementary file 1 [file DataSheet1.pdf]

Supplementary materials

Supplementary Table 1. Collinearity diagnostics

	VIF	TOL	Leamer
Age	1.283	0.779	0.883
ESR	2.287	0.437	0.661
CRP	9.643	0.104	0.322
IgG	1.182	0.846	0.92
IgM	1.044	0.958	0.979
NEU	6.924	0.144	0.38
LYM	5.025	0.199	0.446
NLR	6.194	0.161	0.402
PLR	5.83	0.172	0.414
LMR	2.032	0.492	0.702
PNR	3.9	0.256	0.506
CLR	9.679	0.103	0.321
PIV	2.791	0.358	0.599

ESR, Erythrocyte sedimentation rate; CRP, C-reactive protein; IgG, Immunoglobulin G; IgM, Immunoglobulin M; NEU, Neutrophil; LYM, Lymphocyte; NLR, Neutrophil-to-Lymphocyte Ratio; PLR, Platelet-to-Lymphocyte Ratio; LMR, Lymphocyte-to-Monocyte Ratio; PNR, Platelet-to-Neutrophil Ratio; CLR: C-reactive Protein-to-Lymphocyte Ratio; PIV, Pan-Immune-Inflammation Value; VIF, Variance Inflation Factor; TOL, Tolerance.

Supplementary Table 2. Receiver operating characteristic analysis in overall population

	AUC (95% CI)	Specificity	Sensitivity	Youden index	Cut-off value
Age	0.621 (0.563, 0.679)	0.777	0.424	0.201	43.5
ESR	0.623 (0.566, 0.680)	0.466	0.724	0.19	19.5
CRP	0.636 (0.579, 0.693)	0.43	0.788	0.218	6.825
IgG	0.573 (0.514, 0.632)	0.793	0.359	0.152	15.5
NEU	0.573 (0.514, 0.632)	0.85	0.282	0.132	5.465
NLR	0.638 (0.581, 0.696)	0.513	0.724	0.236	1.88
PNR	0.573 (0.514, 0.632)	0.751	0.4	0.151	54.17
PIV	0.601 (0.543, 0.659)	0.487	0.682	0.169	214.1

AUC, Area under curve; CI, Confidence interval; ESR, Erythrocyte sedimentation rate; CRP, C-reactive protein; IgG, Immunoglobulin G; NEU, Neutrophil; NLR, Neutrophil-to-Lymphocyte Ratio; PNR, Platelet-to-Neutrophil Ratio; PIV, Pan-Immune-Inflammation Value.

Supplementary Table 3. Clinical characteristics and immuno-inflammatory markers of the two groups in pulmonary nodule subgroup

Subgroup analysis	AS (n=193)	AS+PN (n=123)	Z	*P*	$P_{Bonferroni}$
**Demographic/clinical characteristics**					0.05/4=0.0125
Sex (Male, %)	151 (78.2)	91 (74.0)	0.758*	0.384	NSD
Age (Years)	34.0 (27.5, 43.0)	38.0 (30.0, 50.0)	-2.259	0.024	NSD
Smoking (Yes, %)	112 (58.0)	70 (56.9)	0.039*	0.844	NSD
ADCC (Count)	19.0 (0, 25.0)	21.0 (0, 25.0)	-0.119		NSD
**Inflammatory markers**					0.05/3=0.0167
ESR (mm/h)	23.0 (8.0, 41.5)	31.0 (14.0, 58.0)	-3.579	<0.001	SD
RF (IU/mL)	8.0 (6.0, 10.0)	8.0 (6.0, 10.0)	-0.261	0.794	NSD
CRP (mg/L)	8.1 (3.7, 23.4)	16.1 (6.9, 38.2)	-3.955	<0.001	SD
**Immunological indicators**					0.05/4=0.0125
IgG (g/L)	13.0 (11.5, 15.0)	14.0 (12.0, 18.0)	-3.265	0.001	SD
IgA (g/L)	3.0 (2.0, 4.0)	3.0 (2.0, 4.0)	-1.771	0.077	NSD
IgM (g/L)	1.0 (1.0, 2.0)	1.0 (1.0, 25.0)	-3.405	0.001	SD
C3 (g/L)	1.0 (1.0, 1.0)	1.0 (1.0, 2.0)	-0.544	0.586	NSD
**Blood immune cells**					0.05/6=0.008
NEU (× 10^9/L)	4.1 (3.2, 5.1)	4.4 (3.7, 5.7)	-2.774	0.006	SD
LYM (× 10^9/L)	2.1 (1.6, 2.6)	1.9 (1.5, 2.5)	-2.009	0.045	NSD
MON (× 10^9/L)	0.43 (0.35, 0.53)	0.44 (0.36, 0.56)	-0.973	0.331	NSD
EOS (× 10^9/L)	0.12 (0.08, 0.22)	0.12 (0.07, 0.19)	-0.531	0.595	NSD
BAS (× 10^9/L)	0.03 (0.02, 0.05)	0.03 (0.02, 0.05)	-0.589	0.556	NSD
PLT (× 10^9/L)	270.0 (228.5, 322.0)	288.0 (232.0, 347.0)	-1.374	0.169	NSD
**Disease activity/function score**					0.05/4=0.0125
BASDAI (cm)	1.9 (0.8, 2.4)	1.7 (0.9, 2.8)	-0.532	0.594	NSD
BASFI (cm)	1.0 (0.9, 1.8)	1.2 (0.8, 1.9)	-1.105	0.269	NSD
**Composite inflammatory markers**					0.05/4=0.0125
NLR	1.9 (1.5, 2.4)	2.3 (1.8, 3.1)	-4.373	<0.001	SD
PLR	128.1 (98.1, 166.5)	144.4 (111.8, 191.4)	-2.699	0.007	SD
LMR	4.9 (3.9, 6.0)	4.3 (3.4, 5.4)	-3.130	0.002	SD
PNR	66.2 (54.0, 83.7)	59.4 (49.4, 77.8)	-2.345	0.019	NSD
CLR	4.2 (1.6, 12.6)	7.7 (3.4, 19.6)	-4.162	<0.001	SD
PIV	224.4 (140.0, 358.8)	272.2 (194.1, 462.2)	-3.506	<0.001	SD

*, Chi-square value; ADCC, Average daily cigarette consumption; BASDAI, Bath Ankylosing Spondylitis Disease Activity Index; BASFI, Bath Ankylosing Spondylitis Functional Index; ESR, Erythrocyte sedimentation rate; RF, Rheumatoid factor; CRP, C-reactive protein; IgG, Immunoglobulin G; IgA, Immunoglobulin A; IgM, Immunoglobulin M; C3, Complement component 3; NEU, Neutrophil; LYM, Lymphocyte; MON, Monocyte; EOS, Eosinophil; BAS, Basophil; PLT, Platelet; NLR, Neutrophil-to-Lymphocyte Ratio; PLR, Platelet-to-Lymphocyte Ratio; LMR, Lymphocyte-to-Monocyte Ratio; PNR, Platelet-to-Neutrophil Ratio; CLR: C-reactive Protein-to-Lymphocyte Ratio; PIV, Pan-Immune-Inflammation Value.

$P_{Bonferroni}$, 0.05 divided by the number of subsets; SD, Significant difference; NSD, Not significant difference.

Supplementary Table 4. Receiver operating characteristic analysis in pulmonary nodule subgroup

	AUC (95% CI)	Specificity	Sensitivity	Youden index	Cut-off value
Age	0.575 (0.51, 0.641)	0.674	0.472	0.145	39.501
ESR	0.619 (0.557, 0.682)	0.466	0.707	0.174	19.501
CRP	0.632 (0.57, 0.694)	0.43	0.772	0.202	6.825
IgG	0.608 (0.544, 0.672)	0.793	0.398	0.191	15.502
IgM	0.603 (0.545, 0.661)	0.907	0.268	0.175	23.5
NEU	0.593 (0.529, 0.656)	0.850	0.301	0.151	5.465
NLR	0.646 (0.583, 0.708)	0.513	0.724	0.237	1.88
PNR	0.578 (0.514, 0.642)	0.751	0.398	0.150	54.17
PIV	0.617 (0.554, 0.679)	0.523	0.675	0.198	231.385

AUC, Area under curve; CI, Confidence interval; ESR, Erythrocyte sedimentation rate; CRP, C-reactive protein; IgG, Immunoglobulin G; IgM, Immunoglobulin M; NEU, Neutrophil; NLR, Neutrophil-to-Lymphocyte Ratio; PNR, Platelet-to-Neutrophil Ratio; PIV, Pan-Immune-Inflammation Value.

Supplementary Table 5. Clinical characteristics and immuno-inflammatory markers of the two groups in COPD subgroup

Subgroup analysis	AS (n=193)	AS+COPD (n=47)	Z	*P*	$P_{Bonferroni}$
**Demographic/clinical characteristics**					0.05/4=0.0125
Sex (Male, %)	151 (78.2)	39 (83.0)	0.515*	0.473	NSD
Age (Years)	34.0 (27.5, 43.0)	46.0 (35.0, 62.0)	-5.105	<0.001	SD
Smoking (Yes, %)	112 (58.0)	38 (80.9)	8.398*	0.004	NSD
ADCC (Count)	19.0 (0, 25.0)	23.0 (20.0, 26.0)	-2.516	0.012	SD
**Inflammatory markers**					0.05/3=0.0167
ESR (mm/h)	23.0 (8.0, 41.5)	36.0 (20.0, 57.0)	-2.842	0.004	SD
RF (IU/mL)	8.0 (6.0, 10.0)	9.0 (8.0, 11.0)	-2.877	0.004	SD
CRP (mg/L)	8.1(3.7, 23.4)	18.3 (8.0, 38.9)	-3.117	0.002	SD
**Immunological indicators**					0.05/4=0.0125
IgG (g/L)	13.0 (11.5, 15.0)	13.0 (11.0, 16.0)	-0.391	0.696	NSD
IgA (g/L)	3.0 (2.0, 4.0)	2.0 (2.0, 3.0)	-2.304	0.021	NSD
IgM (g/L)	1.0 (1.0, 2.0)	1.0 (1.0, 2.0)	-0.377	0.706	NSD
C3 (g/L)	1.0 (1.0, 1.0)	1.0 (1.0, 1.0)	-0.931	0.352	NSD
**Blood immune cells**					0.05/6=0.008
NEU (× 10^9/L)	4.1 (3.2, 5.1)	4.2 (3.3, 5.4)	-0.464	0.643	NSD
LYM (× 10^9/L)	2.1 (1.6, 2.6)	1.8 (1.2, 2.2)	-2.898	0.004	SD
MON (× 10^9/L)	0.43 (0.35, 0.53)	0.42 (0.33, 0.54)	-0.158	0.874	NSD
EOS (× 10^9/L)	0.12 (0.08, 0.22)	0.14 (0.07, 0.24)	-0.728	0.467	NSD
BAS (× 10^9/L)	0.03 (0.02, 0.05)	0.04 (0.02, 0.05)	-0.809	0.419	NSD
PLT (× 10^9/L)	270.0 (228.5, 322)	251.0 (214.0, 306.0)	-1.791	0.073	NSD
**Disease activity/function score**					0.05/4=0.0125
BASDAI (cm)	1.7 (0.8, 2.4)	1.0 (0.9, 1.8)	-0.032	0.975	NSD
BASFI (cm)	1.0 (0.9, 1.8)	1.8 (0.8, 2.4)	-1.828	0.068	NSD
**Composite inflammatory markers**					0.05/4=0.0125
NLR	1.9 (1.5, 2.4)	2.4 (1.5, 3.6)	-2.520	0.012	SD
PLR	128.1 (98.1, 166.5)	146.2 (113.3, 197.4)	-2.311	0.021	SD
LMR	4.9 (3.9, 6.0)	4.0 (3.0, 5.9)	-2.636	0.008	SD
PNR	66.2 (54.0, 83.7)	59.2 (45.7, 85.0)	-1.265	0.206	NSD
CLR	4.2 (1.6, 12.6)	11.9 (4.1, 25.9)	-3.607	<0.001	SD
PIV	224.4 (139.9, 358.8)	268.8 (159.2, 484.2)	-1.283	0.200	NSD

*, Chi-square value; ADCC, Average daily cigarette consumption; BASDAI, Bath Ankylosing Spondylitis Disease Activity Index; BASFI, Bath Ankylosing Spondylitis Functional Index; ESR, Erythrocyte sedimentation rate; RF, Rheumatoid factor; CRP, C-reactive protein; IgG, Immunoglobulin G; IgA, Immunoglobulin A; IgM, Immunoglobulin M; C3, Complement component 3; NEU, Neutrophil; LYM, Lymphocyte; MON, Monocyte; EOS, Eosinophil; BAS, Basophil; PLT, Platelet; NLR, Neutrophil-to-Lymphocyte Ratio; PLR, Platelet-to-Lymphocyte Ratio; LMR, Lymphocyte-to-Monocyte Ratio; PNR, Platelet-to-Neutrophil Ratio; CLR: C-reactive Protein-to-Lymphocyte Ratio; PIV, Pan-Immune-Inflammation Value.

$P_{Bonferroni}$, 0.05 divided by the number of subsets; SD, Significant difference; NSD, Not significant difference.

Supplementary Table 6. Receiver operating characteristic analysis in COPD subgroup

	AUC (95% CI)	Specificity	Sensitivity	Youden index	Cut-off value
AGE	0.74 (0.661, 0.82)	0.777	0.574	0.352	43.505
ESR	0.634 (0.547, 0.72)	0.813	0.426	0.239	48.501
RF	0.635 (0.55, 0.719)	0.554	0.681	0.235	8.506
CRP	0.647 (0.559, 0.735)	0.611	0.66	0.271	12.94
IGA	0.605 (0.52, 0.689)	0.601	0.617	0.218	2.476
NLR	0.619 (0.516, 0.721)	0.803	0.468	0.271	2.565

AUC, Area under curve; CI, Confidence interval; ESR, Erythrocyte sedimentation rate; CRP, C-reactive protein; IgG, Immunoglobulin G; IgM, Immunoglobulin M; NEU, Neutrophil; NLR, Neutrophil-to-Lymphocyte Ratio; PNR, Platelet-to-Neutrophil Ratio; PIV, Pan-Immune-Inflammation Value.

Supplementary Table 7. Clinical characteristics and immuno-inflammatory markers of the two groups (sensitivity analysis).

Sensitivity analysis	AS (n=193)	AS+PD (n=170)	Z	*P*	$P_{Bonferroni}$
**Demographic/clinical characteristics**					0.05/4=0.0125
Sex (Male, %)	151 (78.2)	130 (76.5)	0.162*	0.688	NSD
Age (Years)	34.0 (27.5, 43.0)	40.5 (31, 52.3)	-3.977	<0.001	SD
Smoking (Yes, %)	112 (58.0)	108 (63.5)	1.144*	0.285	NSD
ADCC (Count)	19.0 (0, 25.0)	22.0 (0, 25.0)	-1.175	0.240	NSD
**Inflammatory markers**					0.05/3=0.0167
ESR (mm/h)	22.0 (8.0, 40.5)	33 (16, 57)	-4.258	<0.001	SD
RF (IU/mL)	8.0 (6.0, 10.0)	8 (6.3, 10)	-1.114	0.265	NSD
CRP (mg/L)	8.4 (3.75, 23.5)	17.8 (7.3, 38.5)	-4.622	<0.001	SD
**Immunological indicators**					0.05/4=0.0125
IgG (g/L)	13.0 (12.0, 15.0)	14 (12, 17)	-2.504	0.012	SD
IgA (g/L)	3.0 (2.0, 4.0)	3 (2, 4)	-0.100	0.920	NSD
IgM (g/L)	1.0 (1.0, 2.0)	1 (1, 24)	-2.956	0.003	SD
C3 (g/L)	1.0 (1.0, 1.0)	1 (1, 1)	-0.028	0.977	NSD
**Blood immune cells**					0.05/6=0.008
NEU (× 10^9/L)	4.1 (3.2, 5.1)	4.4 (3.6, 5.6)	-2.386	0.017	SD
LYM (× 10^9/L)	2.1 (1.6, 2.6)	1.9 (1.4, 2.4)	-3.033	0.002	NSD
MON (× 10^9/L)	0.43 (0.35, 0.53)	0.44 (0.36, 0.56)	-0.803	0.422	NSD
EOS (× 10^9/L)	0.12 (0.08, 0.22)	0.12 (0.06, 0.2)	-0.368	0.713	NSD
BAS (× 10^9/L)	0.03 (0.02, 0.05)	0.03 (0.02, 0.05)	-0.614	0.539	NSD
PLT (× 10^9/L)	270.0 (228.5, 322.0)	275.5 (225.8, 335.3)	-0.203	0.839	NSD
**Disease activity/function score**					0.05/4=0.0125
BASDAI (cm)	1.68 (0.78, 2.44)	1.71 (0.84, 2.54)	-0.891	0.373	NSD
BASFI (cm)	1.0 (0.9, 1.8)	1.1 (0.8, 1.8)	-0.359	0.719	NSD
**Composite inflammatory markers**					0.05/4=0.0125
NLR	1.9 (1.5, 2.4)	2.4 (1.8, 3.3)	-4.682	<0.001	SD
PLR	128.1 (98.1, 166.5)	146.3 (113.6, 192.8)	-3.288	0.001	SD
LMR	4.9 (3.9, 6.0)	4.1 (3.3, 5.4)	-3.896	<0.001	SD
PNR	66.2 (54.0, 83.9)	59.2 (49, 80.4)	-2.337	0.019	NSD
CLR	4.2 (1.6, 12.6)	8.1 (3.4, 19.8)	-4.651	<0.001	SD
PIV	224.4 (139.9, 358.7)	272.4 (188, 467.8)	-3.372	0.001	SD

*, Chi-square value; ADCC, Average daily cigarette consumption; BASDAI, Bath Ankylosing Spondylitis Disease Activity Index; BASFI, Bath Ankylosing Spondylitis Functional Index; ESR, Erythrocyte sedimentation rate; RF, Rheumatoid factor; CRP, C-reactive protein; IgG, Immunoglobulin G; IgA, Immunoglobulin A; IgM, Immunoglobulin M; C3, Complement component 3; NEU, Neutrophil; LYM, Lymphocyte; MON, Monocyte; EOS, Eosinophil; BAS, Basophil; PLT, Platelet; NLR, Neutrophil-to-Lymphocyte Ratio; PLR, Platelet-to-Lymphocyte Ratio; LMR, Lymphocyte-to-Monocyte Ratio; PNR, Platelet-to-Neutrophil Ratio; CLR: C-reactive Protein-to-Lymphocyte Ratio; PIV, Pan-Immune-Inflammation Value.

$P_{Bonferroni}$, 0.05 divided by the number of subsets; SD, Significant difference; NSD, Not significant difference.

Supplementary Table 8. Receiver operating characteristic analysis in overall population (sensitivity analysis).

	AUC (95% CI)	Specificity	Sensitivity	Youden index	Cut-off value
Age	0.621 (0.563, 0.679)	0.777	0.424	0.201	43.5
CRP	0.642 (0.585, 0.699)	0.427	0.794	0.221	6.825
IGG	0.583 (0.518, 0.648)	0.799	0.367	0.166	15.5
NEU	0.573 (0.514, 0.633)	0.85	0.28	0.13	5.465
NLR	0.644 (0.586, 0.702)	0.513	0.732	0.245	1.88
PNR	0.572 (0.512, 0.631)	0.751	0.402	0.154	54.17
PIV	0.604 (0.545, 0.662)	0.508	0.665	0.172	225.72

AUC, Area under curve; CI, Confidence interval; ESR, Erythrocyte sedimentation rate; CRP, C-reactive protein; IgG, Immunoglobulin G; IgM, Immunoglobulin M; NEU, Neutrophil; NLR, Neutrophil-to-Lymphocyte Ratio; PNR, Platelet-to-Neutrophil Ratio; PIV, Pan-Immune-Inflammation Value.

Supplementary Table 9. Results of 5-fold cross validation in the comorbidity of AS and COPD

	Accuracy	AUC	AUC_PR	Precision	Specificity	R^2	MSE	RMSE
**DT**								
CV_1	0.583	0.574	0.224	0.308	0.526	-0.290	0.213	0.461
CV_2	0.792	0.780	0.690	0.615	0.857	0.242	0.150	0.387
CV_3	0.896	0.868	0.484	0.625	0.927	0.290	0.088	0.297
CV_4	0.875	0.905	0.762	0.700	0.921	0.383	0.102	0.319
CV_5	0.792	0.847	0.446	0.412	0.756	0.132	0.108	0.329
**RF**								
CV_1	0.667	0.734	0.314	0.375	0.605	-0.072	0.177	0.420
CV_2	0.771	0.770	0.548	0.571	0.829	0.097	0.178	0.422
CV_3	0.771	0.815	0.338	0.375	0.756	0.111	0.111	0.333
CV_4	0.813	0.841	0.654	0.533	0.816	0.271	0.120	0.347
CV_5	0.792	0.821	0.354	0.400	0.781	0.102	0.112	0.335
**SVM**								
CV_1	0.625	0.582	0.256	0.300	0.632	-0.084	0.179	0.423
CV_2	0.729	0.776	0.622	0.500	0.686	0.152	0.167	0.409
CV_3	0.854	0.896	0.532	0.500	0.829	0.217	0.098	0.312
CV_4	0.875	0.884	0.665	0.667	0.895	0.286	0.118	0.343
CV_5	0.813	0.669	0.246	0.375	0.878	-0.090	0.136	0.369
**XGBoost**								
CV_1	0.688	0.605	0.254	0.353	0.711	0.001	0.165	0.406
CV_2	0.771	0.802	0.647	0.556	0.771	0.221	0.154	0.392
CV_3	0.875	0.826	0.472	0.556	0.902	0.124	0.109	0.330
CV_4	0.813	0.818	0.568	0.546	0.868	0.234	0.126	0.356
CV_5	0.813	0.840	0.443	0.429	0.805	0.153	0.106	0.325

AUC, Area under the curve; AUC_PR, AUC for precision-recall; CV, Cross validation; DT, Decision tree; MSE, mean square error; RMSE, root mean square error; RF, Random forest; SVM, Support Vector Machine; XGBoost, eXtreme Gradient Boosting.

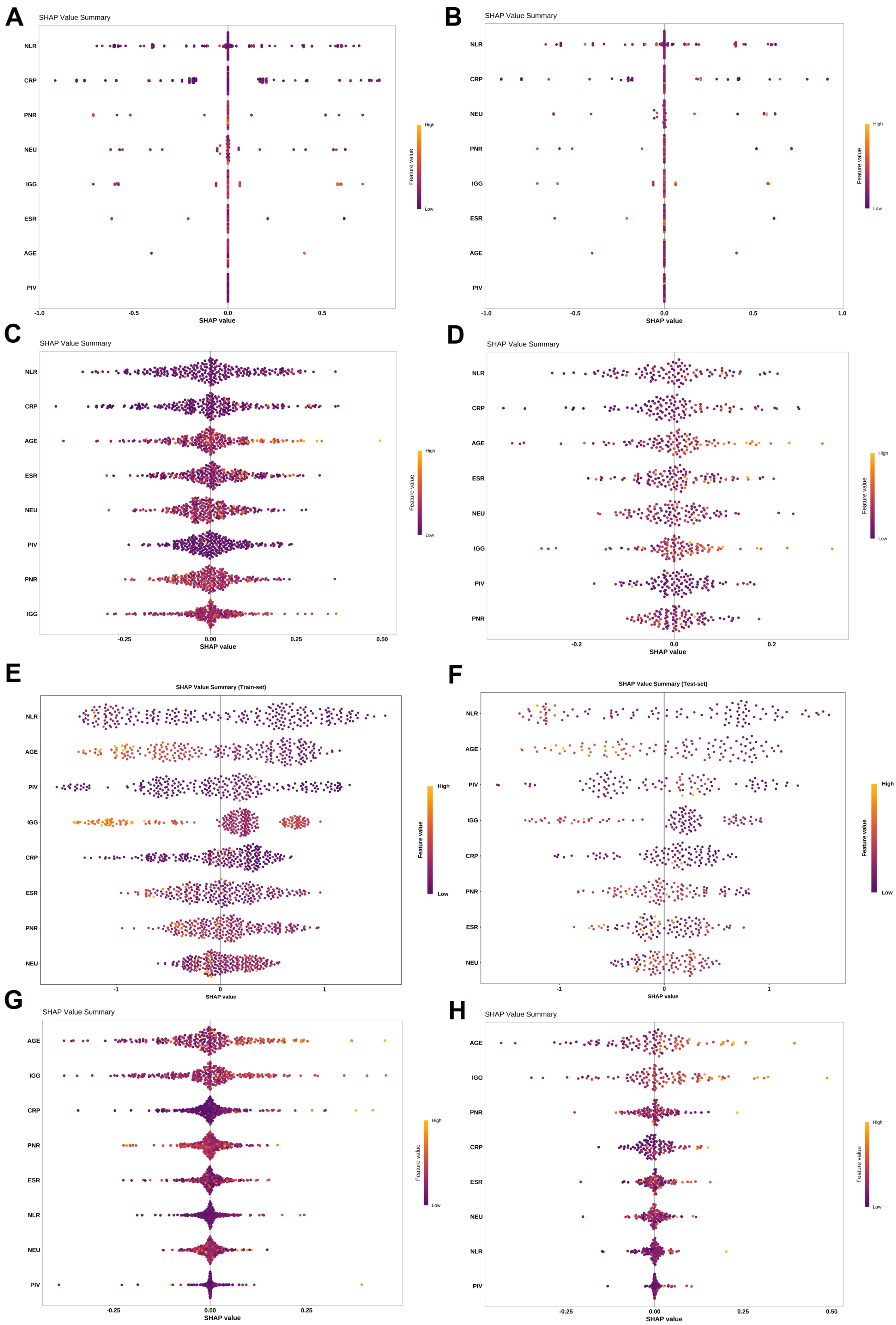

Supplementary Figure 1. SHAP feature importance of ML models for predicting PD risk in AS patients. **(A)** training set and **(B)** test set of the decision tree model, **(C)** training set and **(D)** test set of the random forest model, **(E)** training set and **(F)** test set of the XGBoost model, **(G)** training set and **(H)** test set of the SVM model.

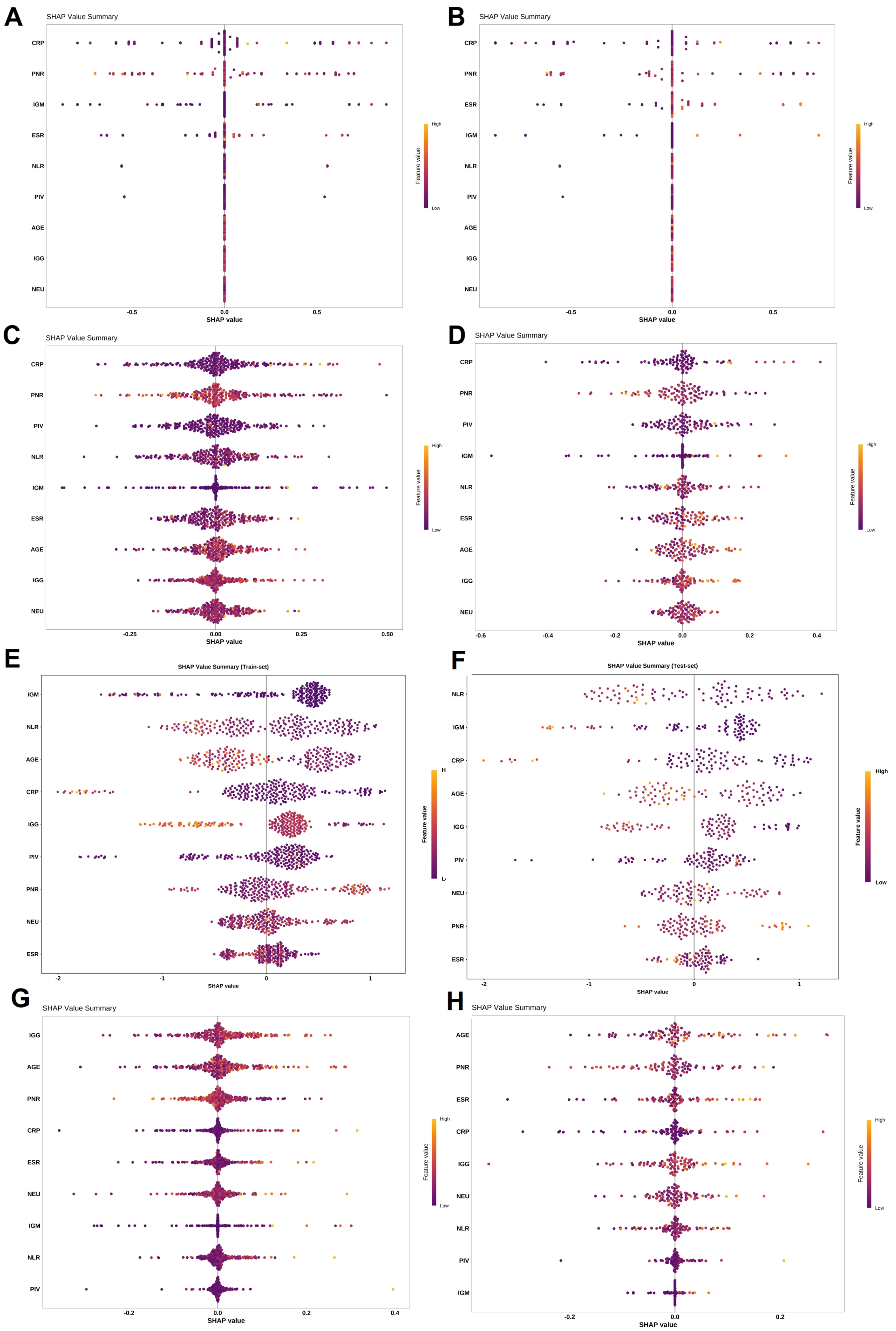

Supplementary Figure 2. SHAP feature importance of ML models for predicting PN risk in AS patients. **(A)** training set and **(B)** test set of the decision tree model, **(C)** training set and **(D)** test set of the random forest model, **(E)** training set and **(F)** test set of the XGBoost model, **(G)** training set and **(H)** test set of the SVM model.

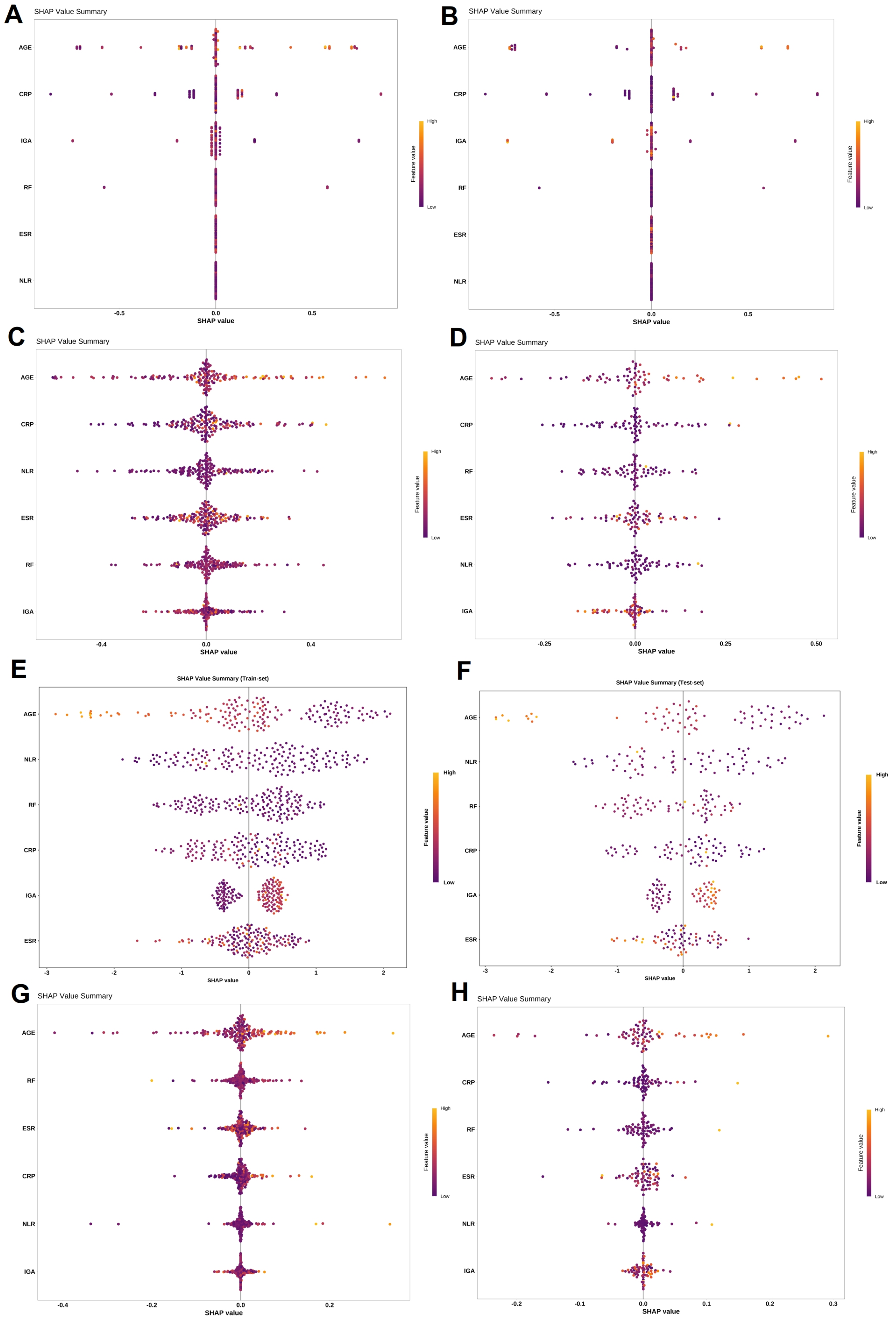

Supplementary Figure 3. SHAP feature importance of ML models for predicting COPD risk in AS patients. (A) training set and **(B)** test set of the decision tree model, **(C)** training set and **(D)** test set of the random forest model, **(E)** training set and **(F)** test set of the XGBoost model, **(G)** training set and **(H)** test set of the SVM model.

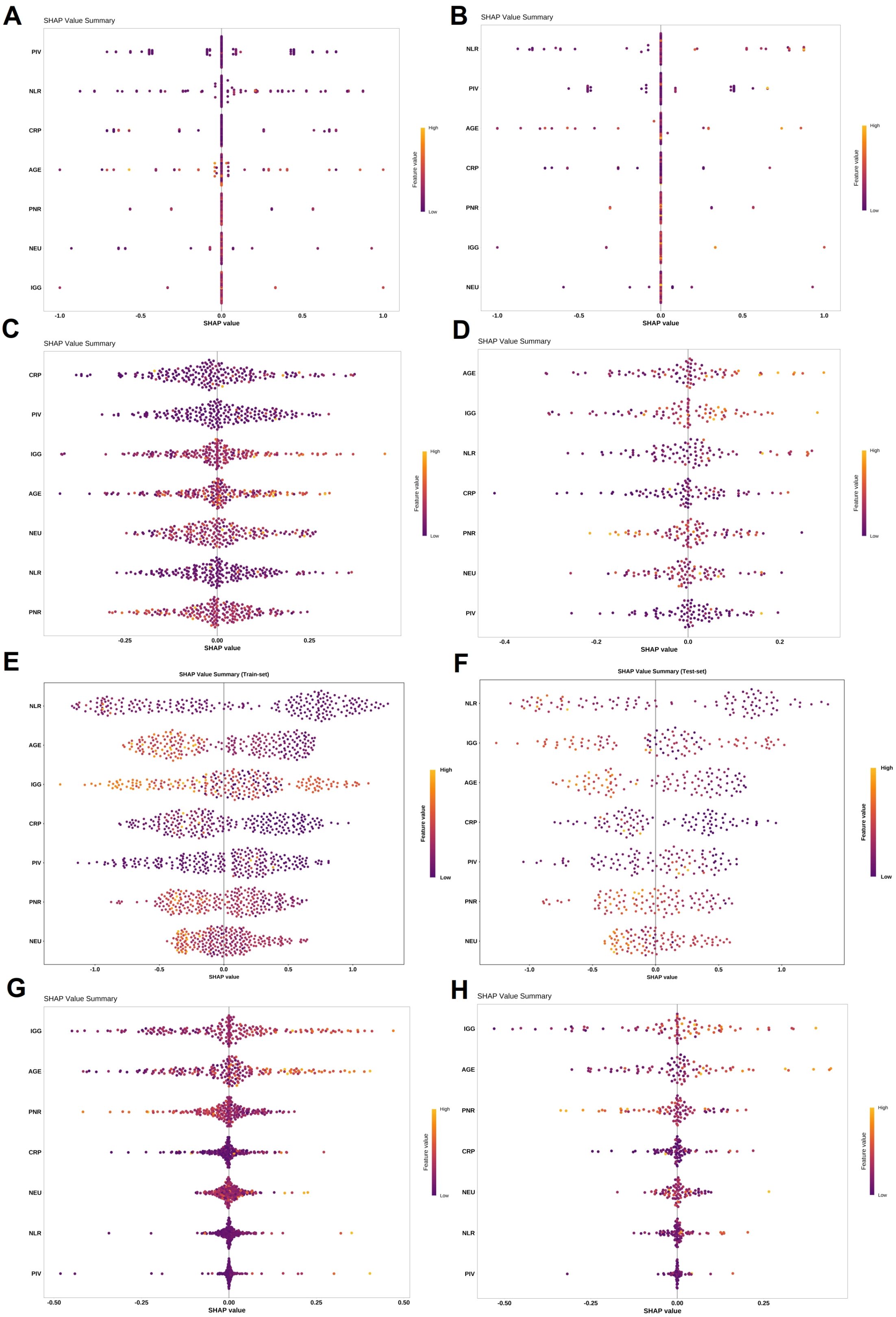

Supplementary Figure 4. SHAP feature importance of ML models for predicting PD risk in AS patients (sensitivity analysis). **(A)** training set and **(B)** test set of the decision tree model, **(C)** training set and **(D)** test set of the random forest model, **(E)** training set and **(F)** test set of the XGBoost model, **(G)** training set and **(H)** test set of the SVM model.
